# Detailed simulation of cancer exome sequencing data reveals differences and common limitations of variant callers

**DOI:** 10.1186/s12859-016-1417-7

**Published:** 2017-01-03

**Authors:** Ariane L. Hofmann, Jonas Behr, Jochen Singer, Jack Kuipers, Christian Beisel, Peter Schraml, Holger Moch, Niko Beerenwinkel

**Affiliations:** 1 Department of Biosystems Science and Engineering, ETH Zurich, Mattenstr, Basel, 26, 4058 Switzerland; 2 Swiss Institute of Bioinformatics, Mattenstr, Basel, 26, 4058 Switzerland; 3 Institute for Surgical Pathology, University Hospital Zurich, Schmelzbergstrasse 12, Zurich, 8091 Switzerland

**Keywords:** SNV, Variant calling, Cancer genomics, Exome sequencing, Variant caller integration

## Abstract

**Background:**

Next-generation sequencing of matched tumor and normal biopsy pairs has become a technology of paramount importance for precision cancer treatment. Sequencing costs have dropped tremendously, allowing the sequencing of the whole exome of tumors for just a fraction of the total treatment costs. However, clinicians and scientists cannot take full advantage of the generated data because the accuracy of analysis pipelines is limited. This particularly concerns the reliable identification of subclonal mutations in a cancer tissue sample with very low frequencies, which may be clinically relevant.

**Results:**

Using simulations based on kidney tumor data, we compared the performance of nine state-of-the-art variant callers, namely deepSNV, GATK HaplotypeCaller, GATK UnifiedGenotyper, JointSNVMix2, MuTect, SAMtools, SiNVICT, SomaticSniper, and VarScan2. The comparison was done as a function of variant allele frequencies and coverage. Our analysis revealed that deepSNV and JointSNVMix2 perform very well, especially in the low-frequency range. We attributed false positive and false negative calls of the nine tools to specific error sources and assigned them to processing steps of the pipeline. All of these errors can be expected to occur in real data sets. We found that modifying certain steps of the pipeline or parameters of the tools can lead to substantial improvements in performance. Furthermore, a novel integration strategy that combines the ranks of the variants yielded the best performance. More precisely, the rank-combination of deepSNV, JointSNVMix2, MuTect, SiNVICT and VarScan2 reached a sensitivity of 78% when fixing the precision at 90%, and outperformed all individual tools, where the maximum sensitivity was 71% with the same precision.

**Conclusions:**

The choice of well-performing tools for alignment and variant calling is crucial for the correct interpretation of exome sequencing data obtained from mixed samples, and common pipelines are suboptimal. We were able to relate observed substantial differences in performance to the underlying statistical models of the tools, and to pinpoint the error sources of false positive and false negative calls. These findings might inspire new software developments that improve exome sequencing pipelines and further the field of precision cancer treatment.

**Electronic supplementary material:**

The online version of this article (doi:10.1186/s12859-016-1417-7) contains supplementary material, which is available to authorized users.

## Background

The detection of genomic variation via sequencing of tumor DNA from cancer patients has become a cornerstone of cancer research. More recently, sequencing-based patient stratification also entered clinical procedures in order to select the best treatment for a cancer patient, e.g. in melanoma [1], colorectal cancer [2], lung cancer [3] and ovarian cancer [4]. Particularly in cases where drug administration depends on the presence or absence of specific genomic variants, it is essential to have robust and sensitive bioinformatics pipelines for variant detection.

However, the computational analysis of sequencing data is challenging. Germline mutations have been inherited from the parents and therefore occur at a frequency of 50% or 100% in virtually every cell. Therefore, with sufficient coverage, germline mutations are relatively easy to detect. In contrast, tumors consist of several genetically distinct subclones, a phenomenon called intra-tumor heterogeneity [5]. Therefore, somatic mutations acquired during cancer progression occur at variable frequencies. Unfortunately, even very low-frequency variants may be critical for treatment outcome, because (i) it may be sufficient if a small portion of the cells promotes tumor growth, e.g. by producing a growth factor and (ii) drug resistance mutations may already be present in small subclones that expand upon treatment. For instance, it has been shown that subclones can harbor driver mutations which are markers of poor prognosis [6], and that some subclones are able to resist chemotherapy [7]. Therefore, detecting even rare mutations is crucial for improving therapy.

To increase the power to detect low-frequency mutations in protein-coding genes, the Whole Exome Sequencing (WES) protocol was introduced [8]. The general idea is to enrich for DNA fragments that hybridize to probes that cover a large set of known exons of protein-coding genes. In order to determine which genomic variants in protein-coding genes have been accumulated during cancer progression, a large number of studies adopt WES to sequence pairs of cancer and normal tissue, i.e., tissue from the tumor and from surrounding non-cancerous tissue from the same organ.

Here, we compare a large range of bioinformatics tools for genomic variant detection for paired tumor-normal WES data. We carefully designed an evaluation framework based on simulated exome sequencing data derived from real data. While simulated data can never model all properties of real data, many sources of errors arise from the data processing steps prior to the variant calling, such as mapping and filtering, and can therefore be accurately modeled with simulated data.

The importance of improvements in the data processing pipelines has been highlighted in several studies and reviewed in [9]. Li [10] and Roberts et al. [11] called variants on a pair of biological and technical replicates, respectively. Li could attribute all variants detected between these replicates, which are by definition false positives, to mapping artifacts. Roberts et al. noted that many false positives found in this experiment had high scores. Therefore, mapping artifacts may severely contaminate any WES analysis even when stringent filters are applied [11]. Simulated data allows us to investigate mapping and alignment post-processing artifacts in detail with the goal to improve the development of WES analysis pipelines.

**Related work** Several recent surveys and reviews on variant calling pipelines have sought to evaluate the increasing number of variant callers, mostly addressing the problem of germline mutation calling [12–15]. Only few studies consider the specific challenges of paired tumor-normal variant calling in cancer, where mixed samples are analyzed. Among those, some comparisons use real data and either solely analyze concordance between tools [16] or evaluate predictions on relatively small sets of validated mutations [13, 17–19]. Kim and colleagues [20] benchmark four anonymous callers on cancer exome sequencing data in order to provide guidelines on how to compare variant callers. The authors analyze discrepancies and concordances between the callers. They assess different ways of validating the mutations, for example by re-sequencing a subset of the variant calls at higher depth. The authors conclude that it might be misleading to base the performance on a small set of validated mutations, since the number of false negatives could be underestimated, and the selection of which mutations were validated could be biased towards one caller. They suggest ranking the mutations according to the callers confidence scores in order to allow for a more comprehensive comparison with different precision cutoffs. Finally, the authors mention that combining several variant callers is another interesting challenge which needs to be addressed. Spencer et al. [21] assess the performance of variant callers to detect low-frequency mutations by creating a mixture of DNA from well-characterized cell lines. However, it is difficult to attribute discordances between tools [11, 22] to the specific sources of errors. Alioto et al. [23] compared the performance of different analysis pipelines for whole-genome cancer sequencing data. The set of ground truth mutations was generated using variants detected in the same matched tumor and normal sample, but at a higher coverage of approximately 300×. They report precision and recall of different analysis pipelines, however it is also difficult to determine the sources of false positive and false negative calls. The authors recommend optimizing the aligner/variant caller combination, and to combine several variant callers. Furthermore, they propose additional comparison studies to also assess, for example, the effect of normal contamination and subclonality.

To address the aformentioned challenges, here we identify error sources in variant calling arising from the bioinformatics pipeline for read alignment and processing. We carefully designed a simulation study based on one diploid normal and eight diploid cancer genomes, where we introduced variants found in a real tumor-normal sample pair (clear cell renal cell carcinoma). This setup allows us to observe a large range of errors introduced during the various processing steps, and to evaluate the performance at different variant allele frequencies, coverages and contamination levels. We classified errors into different categories and could, in some instances, relate the appearance of certain types of errors to the statistical model of a variant caller. We also developed a new combination strategy to combine several mutation callers.

## Results

**Fig. 1:**
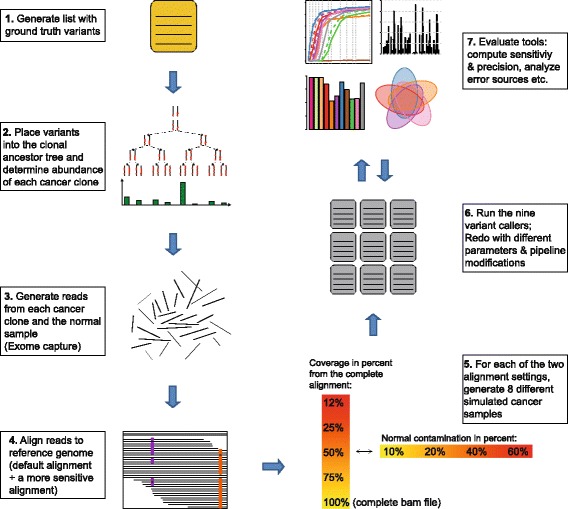
Workflow of the comparison of the nine variant callers. A heterogeneous cancer sample is simulated based on a real renal cell carcinoma sample (steps 1, 2 and 3). Two different alignment settings and eight different coverage and normal contamination levels are employed (steps 4 and 5). The variant callers deepSNV, GATK HP, GATK UG, JointSNVMix2, MuTect, SAMtools, SiNVICT, somaticSniper and VarScan2 are run on all bam files (step 6). The performance of the different tools is evaluated and analyzed in detail (step 7). The tools are also assessed when using various pipeline or parameter modifications as described in Section “Pipeline and parameter improvements”. A more detailed description of the pipeline and the evaluation procedure can be found in the Methods Section as well as in Additional file 1: Section B, and Additional file 1: Section C

We compared the nine somatic variant calling programs deepSNV [24], Genome Analysis Toolkit (GATK) HaplotypeCaller (HP) [25–27], GATK UnifiedGenotyper (UG) [25–27], JointSNVMix2 [28], MuTect [29], SAMtools [10], SiNVICT [30], SomaticSniper [31], and VarScan2 [32]. Figure 1 illustrates the workflow for the comparison in a flowchart. A heterogeneous cancer sample was simulated based on a real renal cell carcinoma sample. The different tools were evaluated and analyzed in detail. First, the performance of all tools with default parameters was assessed as a function of variant allele frequency, coverage and normal contamination. Subsequently, the most prevalent error sources for two different alignment settings were analyzed. Furthermore, the performance of the tools was compared when applying two pipeline modifications. Moreover, five tools were selected for further analysis of the effect of changing parameters. Finally, two combination strategies for variant callers were evaluated, including our newly developed rank-combination, which is implemented in R and available at [33]. A detailed description of the pipeline and evaluation procedure can be found in the Methods Section, as well as in Additional file 1: Section B, and Section C. In the following, we discuss general concepts of somatic variant calling. The statistical models of the tools are described in Additional file 1: Section D.

### Statistical models for somatic variant calling

All variant callers considered here take as input DNA-fragments (reads) aligned to a reference genome. Each discrepancy between the reference and the aligned read could in principle originate from a real change in the genome or from technical artifacts, including sequencing and alignment errors. Since the mechanisms generating true genomic variants are complicated and vary substantially between tumors and cancer types, most somatic variant callers focus on modelling the error sources. The more accurate the different error sources are modeled, the easier it is to identify discrepancies that are unlikely to originate from these error sources and are therefore likely true genomic variants.

In cancer research, we are often interested in the changes between tumor and matched normal samples. The most common approach is to fit a statistical model to the data of both samples and then compute the likelihood. Most sources of errors will be shared between the two samples and will therefore not give rise to significant changes. Remaining artifacts are often difficult to distinguish from true somatic variants. To understand these difficulties, it is insightful to have a closer look at the different statistical models for variant calling. A detailed introduction of the statistical models of all considered variant callers can be found in Additional file 1: Section D. The commands that were used to run the callers in default mode are provided in Additional file 1: Section E.

**Sequencing data pipelines** The outline of a typical WES data processing pipeline is as follows. Sequencing platforms provide a so-called base quality for each position of a read, which quantifies the confidence that the called nucleotide at this position is correct. After the optional step of quality trimming, where positions of reads with low base quality are removed, reads are aligned to the genome with genomic aligners like bowtie2 [34] or bwa [35]. Afterwards, fragments that align to multiple locations (multi-mappers) need to be resolved. The two most common strategies are: (i) deciding for the mapping with highest score (“best”) or (ii) removing all mappings for a fragment as soon as there is more than one (“unique”). Choosing the “best” alignment is often arbitrary if the number of mismatches is identical. If we have decided to select one out of several alignments, the information of whether there were additional alignments and how close their score was to the best alignment should be reflected in the mapping quality score of the selected alignment. The more conservative strategy of selecting “unique” alignments is identical to “best” with a stringent cutoff on the mapping quality.

After running one or more variant callers on the pair of tumor and normal aligned reads, confidence cutoffs have to be defined depending on the requirements of the downstream analysis.

### WES data simulation

A tumor sample is composed of several genetically distinct subclones [5]. In order to generate a realistic scenario for somatic variant calling of tumor samples with intra-tumor heterogeneity, we explicitly generated eight diploid simulated cancer genomes (clones) and one diploid normal genome. Variants detected in a WES tumor-normal sample pair of human clear cell renal cell carcinoma (ccRCC) were placed into these genomes using the software library SeqAn [36]. Variants detected only in the normal sample were placed into the normal genome, equally likely as homozygous or heterozygous mutations. All cancer genomes inherit the normal variants. The cancer clones are related by a clonal ancestry tree, shown in Additional file 1: Figure A. Variants detected only in the real tumor sample are randomly assigned to one of the nodes of the tree. All children of this node inherit the variant. A total of 217,507 somatic and 456,680 germline mutations found in the real ccRCC and its matched normal sample were put in the phylogenetic tree.

Finally, reads are generated from the normal genome and the clones using the software library SeqAn [36] and Wessim [37]. The tumor sample was generated by mixing the reads from the clones and varying proportions of reads from the normal sample. The weighting of the clones was determined by drawing a Dirichlet-distributed random vector.

It is important to note that the simulation includes SNVs and indels, but no copy number variants (CNVs) or aneuploidies. CNVs and aneuploidy also play important roles in tumor evolution. This study focuses on tools for somatic SNV detection. The loss or gain of a part or even a whole chromosome in a subclone, would influence the variant allele frequency of a mutation. Here, the whole spectrum of variant allele frequencies in the interval (0,1] was analyzed, which is why CNVs and aneuploidies do not change the conclusions made. To demonstrate that this is the case, the effect on the performance of the tools in the presence of CNVs and aneuploidies was examined on a subset of the data, and the results are described in Additional file 1: Section F. The results are in line with those from the original simulation.

In the following, we analyze the performance of the variant callers with default parameters for different parameter settings of the alignment. We evaluated the performance of all callers against the simulated ground truth. All cutoffs we apply on the precision of the tools are with respect to the ground truth. We take the tools’ score or confidence values only into account to rank the predictions. Also, the variant allele frequencies are always computed with respect to the the ground truth.

Figure 1 summarizes the worklow for the simulation and evaluation procedure. Different alignment settings and coverage levels are assessed. The various coverage levels are all with 20% normal contamination. The different contamination levels are all at 50% coverage, which corresponds to a median coverage of the targeted regions of 106×. Unless stated otherwise, the performance measures reported here refer to the 50% coverage and 20% normal contamination bam files generated with the sensitive alignment described in Section “Variant calling pipeline”.

### Performance with default parameters

**Fig. 2:**
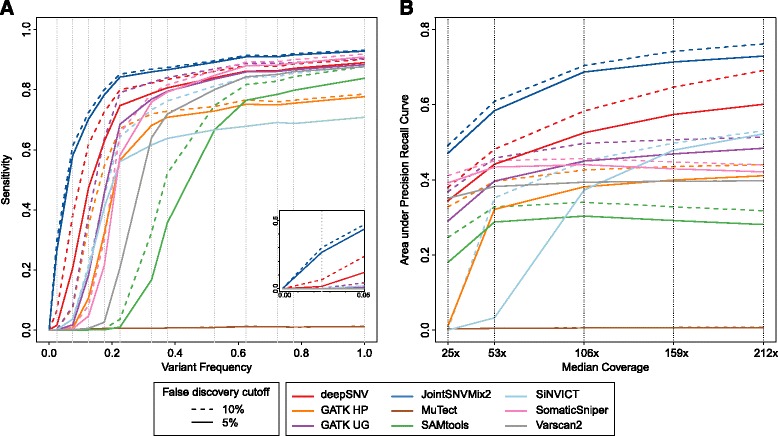
Performance comparison of variant callers with default parameters. **a** Sensitivity of variant callers as a function of the variant allele frequency. To make the predictions comparable we selected the largest set of variants from the top of the list of each caller such that the false discovery rate is still below *α*. We show plots for *α* equal to 0.05 (*solid lines*), and 0.1 (*dashed lines*). If the tool has a very good precision, the two curves for the two *α* cutoffs are identical, as it is the case for MuTect and VarScan2. The details on how the sensitivities are displayed can be found in Additional file 1: Section G. **b** Area under precision recall curve as a function of the coverage. Again the two cutoffs for the false discovery rate *α*=0.05, and *α*=0.1 are chosen (see Additional file 1: Section C). The coverages correspond to the five different levels (12, 25, 50, 75, and 100%) displayed in Fig. 1 in step 5

First, we analyze the sensitivity of the callers as a function of the frequency of the variants (Fig. 2 a). To make the different predictions comparable, we selected the maximal number of variants from the top of the list for each caller, such that the false discovery rate is smaller than a fixed threshold. GATK HP, GATK UG, SAMtools as well as SiNVICT are tools, which report germline and somatic variants. Both types may get a high variant quality score depending on the confidence of the call. Since we are only interested in comparing the performance of somatic variant detection, we filtered out all variants from these four tools that are germline. To this end, we ran the callers separately on the tumor and the normal bam file, and removed all mutations that were found in the normal sample. The resulting filtered variant calls for the tumor sample should contain only somatic mutations. The details on how the sensitivities in Fig. 2 a are displayed can be found in Additional file 1: Section G.

We find substantial differences between the performance of the variant callers, in particular for the sensitivity of low-frequency variants, where deepSNV and JointSNVMix2 clearly show a better performance. For a fixed precision of 90% and for variants with frequencies in the interval (0,0.05), JointSNVMix2 already reaches a sensitivity of 30%, whereas all other tools but deepSNV, with 7%, are close to 0%. For the next highest interval of variant frequencies [0.05,0.1), deepSNV and JointSNVMix2 reach a sensitivity of 38% and 61%, respectively, while the other tools are all still below 9%.

As expected, the more stringent false discovery cutoff of 5% reduces the sensitivity for detection, and especially the low-frequency variants. The two curves for the two false discovery cutoffs of 5 and 10% fall together for VarScan2, because it has a high precision, where the entire set of variants has a precision of 98.7%. MuTect does not provide a continuous quality score, but it reports “PASS” or “REJECT” for each variant. Among the ones with the “PASS” label, the precision is very high: it ranges between 99.7−100%, depending on the coverage. However, it misses many variants, which is reflected in the low sensitivity. The output of SiNVICT does not contain a confidence score for ranking either. However, it does separate the predicted variants into six different lists, which correspond to filters with different stringency levels. Hence, the predicted variants can be ranked according to which of the six lists they occur in. In this case, we had variants which were in the first four lists. Variants in the fourth list not only passed the *p*-value cutoff, but also passed the minimum read depth filter, the strand-bias filter, as well as a filter checking the average position on the reads. Level four had a very high precision of 99.3%. The entire precision-recall curves for all tools can be found in Additional file 1: Figure B. The same performance estimates as displayed in Fig. 2 a were generated when restricting the ground truth variant set to locations with a coverage of at least 25×, which is displayed in Additional file 1: Figure C.

Next, we assessed how the performance of the variant callers depends on the coverage and the contamination of tumor samples with normal tissue. To this end, we generated eight different bam files with various coverage and contamination levels, as illustrated in Fig. 1 step 5. As performance measurement, we use the area under the precision-recall curve of the top predictions which satisfy a precision of at least 90% or 95%, respectively (auPRC_90_ and auPRC_95_, see Additional file 1: Section C). For most tools, the performance rises substantially when increasing the median coverage from 25× to 106× (Fig. 2 b). However, with a coverage of above 106× the performance of most tools saturates, or even decreases.

Increasing the contamination with DNA fragments from the non-cancer cells does lead to a decrease in performance, which can be explained by the expected loss of power (Additional file 1: Figure D). However, the decrease is relatively mild. Variants that are present at higher frequencies are still detectable even with a high rate of normal contamination.

In order to obtain a measure of the variability of the results, 50% subsampling from step 5 in Fig. 1 was repeated ten times, with subsequent variant calling and evaluation. Additional file 1: Figure E displays the area under precision recall curve (auPRC) when restricting to a precision of at least 95 and 90%. The performance estimates of the tools are very stable.

### Analysis of error sources

**Fig. 3:**
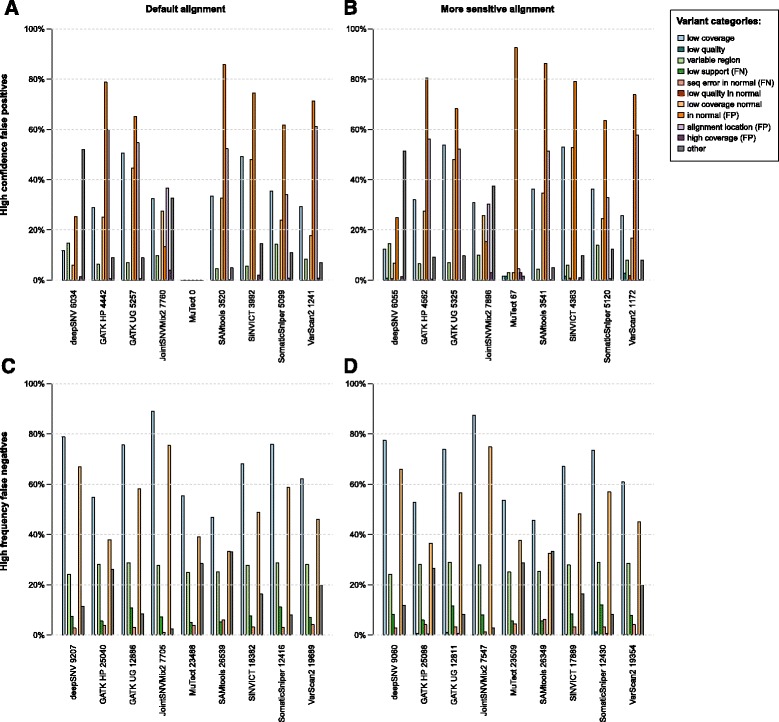
Categories of variant calling errors depending on the quality of the alignment. The top panel (**a** and **b**) shows the error categories for the high confidence false positives (prediction sets with at least 95% precision). The bottom panel (**c** and **d**) shows the error categories for the high-frequency false negatives (ground truth allele frequency ≥25%). The left panel (**a** and **c**) displays the error sources when running **default bowtie2 alignments**, and the right panel (**b** and **d**) displays the error sources when running **more sensitive alignments**, which were performed with parameters —very-sensitive -k 20, and then choosing the primary alignment for each read with several alignments (samtools view -F 256), i.e. the “best” option. The definition of the categories can be found in Section “Analysis of error sources”

We categorized high-confidence false positive predictions, defined as false positives that are in the variant set when restricting it to a precision of at least 95% (Fig. 3 a, 3 b) and high-frequency false negatives (frequency ≥25%; Fig. 3 c, 3 d) into groups of likely error sources. These sets of false positive and false negative predictions are especially interesting to examine. The false positives that are high-confidence, i.e. highly ranked by the tool, are the ones that would likely remain after filtering according to the quality score or *p*-value. Concerning the false negatives, it is clear that low-frequency variants are much more difficult to distinguish from sequencing and alignment artifacts. However, one would expect to detect the variants at higher frequencies. The analysis of these sets of false variant calls sheds light on how the pipeline could be improved.

We define the following error categories: If the coverage at the variant loci was less than 25× reads in the cancer sample, the category *low coverage* applies. The error source *low quality* signifies that the maximal mapping quality of a read supporting the variant was below 31. The category *variable region* denotes that at least one indel or more than 4 SNVs were within 10 bp distance of the variant. The class *low support* represents loci with sufficient coverage, but the reads that support the variant were not aligned. If there was a sequencing error in the normal sample, which gave the impression that the mutation is germline, the error source *seq error in normal* applies. The category *low quality in normal* signifies that, although the total coverage would be high enough, there were ambiguous alignments with low mapping quality in the normal sample resulting in a lack of power for variant calling. In the case where the coverage was less than 25× in the normal sample, the class *low coverage in normal* applies. The category *in normal* represents the case where the variant was introduced in the normal genome and is therefore a germline mutation. The error source *alignment location* denotes the variant was not reported as soon as the decision for multi-mappers was taken for the correct location instead of the “best”. If the correct location was not among the alignments the read was discarded. In the case that the coverage in the cancer sample was more than 200×, the variant is labelled *high coverage*. The category *other* applies for all variants which cannot be attributed to any of the above-mentioned error classes.

For each error source and each tool, the percentage of variants that fall into the respective error source is displayed in Fig. 3. The total number of false positives or false negatives is stated next to the name of the tool. Since variants can fall into several categories, the precentages of the different error sources do not sum up to 100%. The category *other* however, is exclusive, since it contains all variants that did not fit into any of the specified error sources. Also, the categories *low support*, as well as *low quality in normal* imply that there was sufficient coverage in the tumor or normal sample, respectively. Therefore, variants in these categories cannot be classified at the same time as *low coverage* or *low coverage in normal*, respectively. As mentioned in the flowchart in Fig. 1 step 4, two different alignment settings were chosen: the default alignment and a more sensitive alignment, in which more runtime is invested into accurate alignments.

We observe that a substantial source of false positives and especially false negatives is *low coverage* and *low coverage in normal*. As described above, a variant is classified to reside in a *variable region* if there is an indel or more than 4 SNVs within 10 bp distance. These multiple mismatches or gaps in a small region cause uncertainty in the alignment of reads resulting in false positive and false negative SNV calls (Fig. 3 light green). We assess the effect of local realignment around indels in the next section.

In light of the fact that *low coverage* was a major error source, the coverage profile of the sample was computed in order to ensure that the overall coverage is of good quality. As shown in Additional file 1: Figure F, the coverage is very good. For instance, 98.8% of the targeted regions is covered with 25× reads or more, and 95.7% of the targeted regions is covered with 51× reads or more.

Pertaining to the false positive calls, for most tools, many can be attributed to the category *in normal*. These are variants that are actually germline, and have been erroneously classified as somatic. An additional germline filter for the variants might improve the performance, which is assessed in the next section.

The direct comparison of the default and the more sensitive alignment reveals some effects that the alignment has on the false positives and negatives. Overall, the sensitivity to detect variants increases slightly with the more sensitive alignment. For most tools, the number of true positives is increased, which in turn leads to a higher absolute number of false positives with the same precision cutoff of 95%. The error source *alignment location* is reduced for GATK HP, GATK UG, JointSNVMix2, SAMtools, SomaticSniper and VarScan2 when using the more sensitive alignment. The proportion of variants falling into *low quality* rises for most tools for the more sensitive alignment, but it always remains below 3% (Fig. 3 b, d, dark blue and red). The total number of high-frequency false negatives is reduced for deepSNV, GATK UG, JointSNVMix2, SAMtools, SiNVICT, and VarScan2.

When comparing the error profiles of false negative calls of the tools, it is evident that they are highly correlated. Additional file 1: Figure G displays the correlations of error profiles between the tools. More precisely, the minimum correlation between the error profiles of the false negative calls is 0.79, between JointSNVMix2 and SAMtools, and the maximum correlation is 1.00, between GATK UG and somaticSniper. Concerning the false positive calls, the error profiles of the tools are more diverse. JointSNVMix2 and MuTect even show a slightly negative correlation. Overall, deepSNV and JointSNVMix2 seem to be the least correlated in their false positive error profile with any of the other tools.

### Pipeline and parameter improvements

In this section, we demonstrate the effect on the performance when applying changes to the pipeline or parameters of the tools. Moreover, we assess two combination strategies for variant callers.

**Fig. 4:**
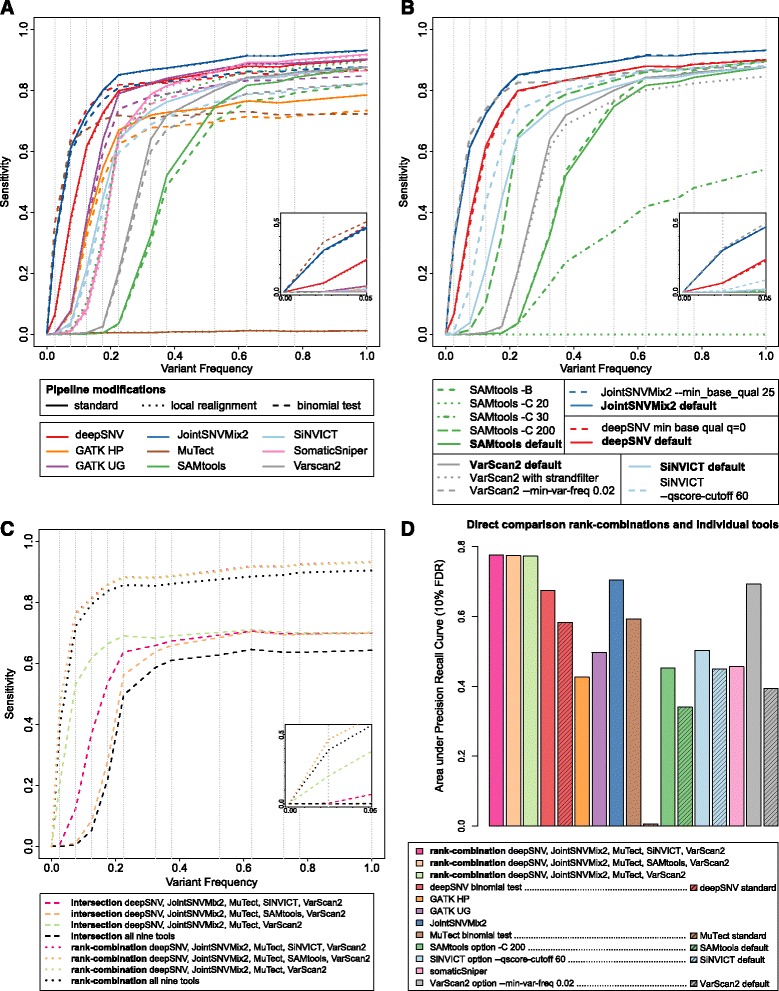
The effect of pipeline modifications, parameter changes, and combination strategies. We show the sensitivity for the prediction set with at least 90% precision. **a** Performance when applying local realignment around indels or the binomial test as a germline filter. **b** Performance of deepSNV, JointSNVMix2, SAMtools, and VarScan2 with different choices of parameters. Additional file 1: Figure H depicts the performance for all parameters that were assessed. **c** Performance of rank-combinations and intersections of calls from several tools. From each tool, we took the best version. In particular, deepSNV and MuTect with the binomial test as germline filter, SAMtools with option -C 200, SiNVICT with –qscore-cutoff 60, VarScan2 with the parameter –min-var-freq 0.02, as well as the default runs from GATK HP, GATK UG, JointSNVMix2, and somaticSniper. **d** Summary barplot displaying the performance of the three best rank-combinations as a comparison to each tool individually. If a tool parameter or pipeline change has been used in the rank-combinations, also the performance of the tool in default mode is shown. The y-axis measures the area under precision-recall curve when allowing a false discovery rate of up to 10% (see Additional file 1: Section C)

**Adressing error sources through pipeline modifications** The analysis of error sources revealed that many SNVs can be attributed to *variable region*, which indicates that the SNV was in a region with indels or a cluster of SNVs. The development team of GATK recommends to use the tool GATK-IndelRealigner as an alignment post-processing step before the variant calling [27]. According to the instructions [38] the tool minimizes the number of mismatches across all reads in regions around insertions and deletions (indels). Here, we assessed whether the GATK-IndelRealigner impacts the performance of variant callers. We note that all predictions are almost identical in sensitivity and precision, except for SAMtools, which benefits greatly (Fig. 4 a, dotted lines).

Another prominent source of false positives is the category *in normal*, which indicates that the variant was actually germline and erroneously classified as somatic. Here, we assess the effect of a post-variant-calling filter that refines the variant calls by filtering out mutations that could likely be germline mutations. More precisely, for each variant loci, the number of reads supporting the variant in the normal sample, as well as the total coverage in the normal sample are taken into account. Assuming a sequencing error rate of at most 0.5%, one can employ a binomial test to assess whether the observed number of variant reads is higher than expected for a sequencing error. If the *p*-value is below 0.05, the variant could likely be a germline mutation and is filtered out. Figure 4 a (dashed lines) depicts the performance of the tools after this post-variant-calling filter. It reduces the sensitivity for all tools but deepSNV and MuTect. For deepSNV, the performance for low-frequency variants is greatly improved, whereas it becomes less sensitive for higher frequencies. With the binomial test, deepSNV reaches a sensitivity of 64% for variants with ground truth frequencies in [0.05,0.1), instead of a sensitivity of 38% without this filter. For Mutect, the sensitivity for variants with ground truth frequencies in [0.15,0.2) was 71% instead of 1% without this post-variant-calling filter.

**Parameter optimization of variant callers** We selected five variant callers to investigate if further performance improvements can be achieved by tuning the parameters of the callers. We chose deepSNV and JointSNVMix2 for their very good overall performance. We assessed the effect of changing the filters for base or mapping quality, because this could have an impact on the performance. Furthermore, we chose SAMtools to explore the effects of certain parameters for which it would be difficult to assign an intuitive value since their impact is unclear. These parameters are -B for recalculation of the base qualities, and -C for recomputing the mapping quality. Moreover, VarScan2 was selected to assess whether a tool that did not perform so well can be improved by a straightforward change of a default parameter: The default threshold for the variant allele frequency is 0.10, which explains the poor performance in the low-frequency range. Also, the impact of applying the strand filter was assessed. Finally, SiNVICT allows setting a threshold for the q-score via the parameter —qscore-cutoff <INT>, which reflects the confidence of the variant call. The q-score is not printed by SiNVICT to the output list of variants, hence a ranking according to this value is not possible. However, adjusting the q-score cutoff threshold with the parameter —qscore-cutoff may lead to an increase in performance.

We found the predictions of deepSNV and JointSNVMix2 to be highly robust against varying thresholds for base and mapping qualities (Fig. 4 b red and blue). The default threshold for the minimum base quality in deepSNV is 25. Lowering this threshold to 0, and thereby also including more potential sequencing errors in the read counts, only leads to a very subtle decrease in performance. Additional file 1: Figure H displays the performance for all parameters that were assessed.

The prediction performance of SAMtools varies substantially in both directions when choosing different values for the parameter -C <INT> (Fig. 4 b green). According to the manual [39], the parameter -C of SAMtools reduces the effect of reads with an excessive number of mismatches. Varying -C between 20 and 200 resulted in large differences in performance ranging from auPRC_90_=0.00 for -C 20 and auPRC_90_=0.45 for -C 200.

The default minimum variant frequency for VarScan2 for heterozygous mutations is 0.10 according to the manual [40], which hampers detection of low-frequency variants. Setting this threshold to 0.02 yields great performance improvements, especially in the low-frequency range (Fig. 4 b grey). The auPRC_90_ is improved from 0.39 to 0.69. Applying the strand filter leads to a decrease in sensitivity, which could indicate that this filter is too conservative.

Different values for the —qscore-cutoff parameter in SiNVICT lead to an increase or decrease in performance, as shown in Additional file 1: Figure H panel E. The optimal value might depend on factors such as coverage and contamination level. The default value is 95, which is probably best for very high coverage data. In this case, the optimal value was found to be —qscore-cutoff 60. It improves the auPRC_90_ from 0.45 to 0.50.

**Variant caller combination strategies** It has been noted that a combination of variant callers may be beneficial to improve sensitivity and specificity of predictions [9, 41]. One straightforward way of combining variant callers is to take the intersection of several tools. We assessed whether the top predictions of the top five variant callers from the default run according to auPRC_90_ (deepSNV, GATK UG, JointSNVMix2, SiNVICT and SomaticSniper) conform. Additional file 1: Figure I displays the Venn diagrams for three different thresholds for the false discovery rate. The evaluation of the set of variants which were shared between the five tools revealed that the precision is very high, as it ranges between 99.6−100%. However, many variants are missed, e.g. the recall is only at most 42.8%, when restricting the individual tools to a false discovery rate of 10%. This demonstrates that taking the intersection of many tools might be a too conservative choice. Additional file 1: Table S2 lists the number of variants which are shared between all pairs of two variant callers and their auPRC_90_.

Finally, we developed a new method to integrate predictions from multiple callers by combining the ranks of variants across callers (see Additional file 1: Section H). The basic idea is to combine the ranks after having standardized the correlation between the tools. We refer to this approach as the rank-combination. From each tool, we took the best version of the assessed pipeline or parameter settings: That is, deepSNV and MuTect with the binomial test as germline filter, SAMtools with -C 200, SiNVICT with option —qscore-cutoff 60, VarScan2 with the parameter —min-var-freq 0.02, as well as the default runs from JointSNVMix2, GATK UG, GATK HP, and somaticSniper. Figure 4 c demonstrates that the performance of the rank-combination is always better than the intersection of the variants of the same callers. By intersection of tools, we refer to the variants which are shared between the callers. The sensitivity decreases as more tools are considered for the intersection. Interestingly, for the rank-combination of callers, it is better to take more tools. The overall sensitivity when combining all nine tools is 75%, where any individual tool only reaches at most 71% with the same fixed precision of 90%. However, the rank-combination of deepSNV, JointSNVMix2, MuTect, SiNVICT, and VarScan2 performs best, and is also better than the rank-combination of all tools. More specifically, it reaches an overall sensitivity of 78% with a fixed precision of 90%. Especially in the low-frequency range, e.g. for variants with frequencies in the interval (0,0.05), the rank-combination of deepSNV, JointSNVMix2, MuTect, SiNVICT, and VarScan2 outperforms the individual tools by reaching a sensitivity of 46%, where the maximum sensitivity of any of the tools individually is 36%, reached by MuTect with the binomial filter. The rank-combination of deepSNV, JointSNVMix2, MuTect, SAMtools and VarScan2 is almost as good as the one with SiNVICT at a precision of 90%, and at a precision of 95% even slightly better. Figure 4 d summarizes the performances of the three best rank-combinations and all nine individual tools with the area under precision-recall curve when the precision is at least 90%. Additional file 1: Table S1 displays the auPRC_95_ and auPRC_90_ values for the ten best rank-combinations.

## Discussion

Our study on simulated data revealed substantial differences between the tools, and identified possibilities to improve cancer exome sequencing pipelines. The relatively high sensitivity for low-frequency variants of deepSNV and JointSNVMix2 is the result of the explicit modeling of the variant allele frequency in the statistical model of these tools. Neither method assumes cancer variants to have undergone clonal expansion. Hence, the statistical model of deepSNV and JointSNVMix2 seems to be the most appropriate for the read count data obtained from heterogeneous tumor samples. By contrast, other methods, such as GATK HP, GATK UG, and SAMtools assume that the variants are clonal, i.e. either heterozygous with a variant allele frequency of 0.5 or homozygous with a frequency of 1.0. In cancer, however samples are not expected to be monoclonal, but rather a mixture of genetically distinct subclones [5]. Subclonal variants that exist at a low frequency might be very important.

When varying thresholds for base and mapping qualities, the performance of deepSNV and JointSNVMix2 remained very stable. The model of deepSNV takes into account overdispersion which could explain the quite robust performance. The statistical model of JointSNVMix2 explicitly considers base and mapping qualities and therefore is immune to any changes in these thresholds.

With a median coverage of above 106×, the performance of most tools does not show a substantial improvement, or even decreases slightly in the case of SAMtools and somaticSniper. This saturation can be attributed to error sources that cannot be resolved with higher coverage in these models. In contrast, especially deepSNV and SiNVICT always perform better with increasing coverage. This underlines the fact that they were designed and tested for very high coverage data.

Concerning the way of reporting the variants, MuTect and SiNVICT do not provide a confidence score for each variant, in contrast to the other tools. We speculate that the performance would be better for MuTect and SiNVICT, if they reported the confidence score as well. This would allow ranking the variants accordingly, and might lead to higher sensitivities for the same precision cutoff.

Regions with coverage less than 25× in the tumor or normal sample cause many false positives and false negatives. The extent of this source of errors can be reduced by aiming for a high coverage when planning an experiment. However, simply increasing the coverage, e.g. by amplifying more, might not solve the problem entirely, since regions with low coverage could be due to alignment problems. If a genomic region contains an accumulation of somatic or germline mutations, the reads might not align any more to the reference genome. In fact, among the false negatives, 61% of variants that fall into *variable region* are also in the category *low coverage*. This points towards alignment problems in the presence of many mismatches. Approaches that possibly lead to an improvement could be to re-align the unmapped reads while allowing for more mismatches or to locally assemble the haplotypes. The GATK-IndelRealigner showed only a limited effect on the performance of the tools. This might be explained by the relatively small number of indels that were introduced in the simulated cancer sample: Among the introduced mutations, there were 0.67% indels, since this study focuses on SNVs. An approach that extends the idea of the GATK-IndelRealigner to any region with many variants, including SNVs, might be promising. Moreover, the GATK-IndelRealigner decides for a single alignment solution, instead of keeping track of the uncertainty. It could help to enumerate or sample all high-scoring local alignment possibilities. Approaches like the one used by the GATK HaplotypeCaller, which reassembles the reads into haplotypes, are promising. However, reads that did not align at all due to increased variability are not included. Results might be improved if this step would already be incorporated during the alignment. Then reads which would otherwise be discarded as unmapped would be included as well. These suggestions point towards a tight interdependence of alignment and variant calling, which should be treated as a single optimization problem. This is clearly computationally more demanding than current variant calling pipelines, but given that problems cannot be resolved by deeper sequencing, more sophisticated algorithms are necessary.

The analysis of error sources also revealed that among the main confounding factors when calling somatic variants are germline mutations, which are erroneously classified as somatic. The post-variant-calling filter, which removes potential germline mutations, increased the performance for deepSNV and MuTect. This improvement of deepSNV is in line with the fact that the error source *in normal* was the highest except for false positive calls that could only be assigned to *other* error sources. For MuTect, 93% of the false positive calls could be assigned to *in normal*, which explains the great performance improvement with this germline filter. The fact that these two callers had problems with distinguishing germline and somatic mutations could be explained by the underlying model. The method of deepSNV calls a variant if the variant allele frequencies differ significantly in the tumor and normal sample, but there is no threshold on the allowed maximal variant allele frequency in the normal sample. MuTect uses two tests when determining a variant. The first one compares the variant model against an error model using the observed read counts in the tumor sample. The second one considers the read counts in the normal sample to test the possibility of a germline mutation. Therefore, the normal and tumor read counts are not compared directly.

Groups of variant callers that do not have strong correlations in their error profiles are interesting candidates for combination strategies. The analysis of the error sources and the correlations of error profiles revealed that deepSNV and JointSNVMix2 were the least correlated in their false positive error profiles with any of the other tools. MuTect, SiNVICT and VarScan2 were the least correlated with JointSNVMix2. Together with the rank-combination, these five tools reached the overall best auPRC_90_. Interestingly, even though SAMtools is by itself not among the five best callers, it is part of one of the best rank-combinations, which also suggests that the tools complement each other in a synergistic way. This is in line with the fact that JointSNVMix2 and SAMtools are the least correlated among all false negative error profiles. And the false negative error profile of SAMtools was overall the least correlated to the profiles of the other tools.

## Conclusions

Our experiments on simulated data revealed that, with default parameters, deepSNV and JointSNVMix2 outperformed the other methods, especially in detecting low-frequency variants. Furthermore, deepSNV and JointSNVMix2 were fairly robust against changes in the default thresholds for base or mapping qualities, which increases the confidence that these tools will perform equally well on other data sets of unknown quality. MuTect showed very competitive performance for low-frequency variants after applying an additional germline filter, which also further increased the sensitivity of deepSNV. VarScan2 improved substantially when changing a default parameter.

The comparison of the default alignment to the more sensitive alignment demonstrated that the tools in general detect more true SNVs from higher quality alignments. We conclude that it is worthwhile investing more in runtime during the alignment to obtain improved performance. Furthermore, the results that we obtained from analyzing the error sources revealed that it might be beneficial to treat alignment and variant calling as a single optimization problem.

The effect on the performance when varying the parameter -C for SAMtools was very heterogeneous. We suggest that, if this parameter is used, it should not be set to an arbitrary value without the possibility to estimate its effect.

The combination analysis showed that the intersection of tools is in general too conservative. The sensitivity decreases when restricting the variants to be found by more tools. Conversely, the rank-combination approach, where the ranks of the tools are combined after standardizing their correlation, proved to be very promising. The rank-combinations were better than the intersection of the same tools. And most of all, the rank-combination of deepSNV and MuTect with the germline-variant-filter, JointSNVMix2, SiNVICT with —qscore-cutoff 60, and VarScan2 with the parameter —min-var-freq 0.02, performed the best at a precision of 90%, and was better than any of the tools alone.

We conclude that many errors can be avoided by investing runtime into very sensitive alignments and using appropriate statistical models such as deepSNV and JointSNVMix2 or combination strategies such as the rank-combination. However, there is still a great need for improving variant calling and alignment in mixed tumor samples.

## Methods

### Simulation

We simulated cancer and normal read data starting from variants that had been identified in a real tumor-normal pair of clear cell renal cell carcinoma. The samples were obtained from the tissue biobank of the Institute of Surgical Pathology at the University Hospital Zurich. DNA was extracted from frozen sections of the tumor and normal tissues using the Blood and Tissue Kit (Qiagen). The exome was sequenced using the Illumina HiSeq 2000 system. Variants that were found only in the tumor sample were randomly assigned to 8 diploid clones (randomly deciding for each variants zygosity). The relative abundance of the clones was sampled from a Dirichlet distribution with concentration parameter $\frac {1}{8}$ for all clones. We generated 16 DNA sequences by introducing the variants into the hg19 reference DNA sequence and then used Wessim [37] to create artificial reads based on an Illumina error model. For more details, see Additional file 1: Section B. It is important to note that the simulation might influence the extent of certain error sources. However, it is expected that all detected error sources occur in real data sets, possibly with different frequencies, and are therefore important to be addressed.

### Variant calling pipeline

We used bowtie2 [34] for all alignments, with the parameters —very-sensitive and -k 20, and then chose the primary alignment for each read with several alignments using samtools (samtools view -F 256), i.e. the “best” option. We also used the default parameters of bowtie2 as noted in Fig. 3.

We ran all variant callers with default parameters, except for technical parameters that do not influence the model for variant calling. When assessing the effect of different parameters, we indicate which parameters were changed (Fig. 4 b and Additional file 1: Figure H).

### Evaluation

For the evaluation, we distinguish between two types of substitutions. In the first case, the fragment of DNA has been replaced by a another fragment of the same size, i.e. a multi-nucleotide variant (MNV). In the second case, the fragment was replaced by a new fragment of different size, i.e. it also includes an indel. Due to the different reporting behavior of variant callers, the evaluation of variants is challenging. To account for these differences, we first split MNVs into individual SNVs. However, in the second case, the substitution cannot be uniquely split into smaller variants, which makes it more difficult to evaluate independently of the reporting behaviour of a tool. For example, one tool may report a variant *ACGG →GCGGG* at position *i*, while another tool reports variant *A →G* at position *i* and *GG →GGG* at position *i*+2.

To generate the ground truth SNVs from the true cancer sample, we used the tool Freebayes [42], which is not part of the comparison. Freebayes has a strong tendency to merge nearby variants. To make sure that predicted SNVs that are correct, but reported differently in the ground truth, are not penalized, we filtered out all predictions that overlap with a ground truth substitution that contains an indel. These substitutions were not counted when computing the performance estimates. However, on average, 98.9% of predictions unambiguously match with no or exactly one ground truth variant and are therefore unambiguous to evaluate.

## Additional file

Additional file 1 Supplemental methods and results [43–53]. (PDF 1187 kb)

## Abbreviations

auPRC: Area under precision-recall curve
ccRCC: Clear cell renal cell carcinoma
CNV: Copy number variant
EM: Expectation-maximization
FDR: False discovery rate
GATK: Genome Analysis Toolkit
HP: HaplotypeCaller
LOH: Loss of heterozygosity
MAP: Maximum a posteriori probability
MNV: Multi-nucleotide variant
SNV: Single-nucleotide variant
UG: UnifiedGenotyper
VAF: Variant allele frequency
VCF: Variant call format
WES: Whole exome sequencing
