# Supplementary material for: Detailed simulation of cancer exome sequencing data reveals differences and common limitations of variant callers
Source: BMC Bioinformatics. 2017 Jan 3;18:8. doi: 10.1186/s12859-016-1417-7 (PMC5209852; doi:10.1186/s12859-016-1417-7)
Supplement: Additional file 1 — Supplemental methods and results [43–53]. (PDF 1187 kb) [file 12859_2016_1417_MOESM1_ESM.pdf]

Hofmann et al.: Detailed simulation of cancer exome sequencing data reveals differences and common limitations of variant callers

Additional file 1

Additional File 1
Supplemental methods and results

Additional file 1: Section A (Figures)

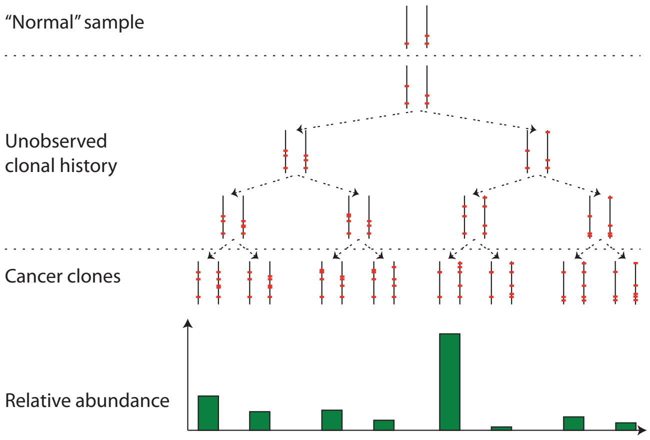

Additional file 1: Figure A: **Illustration of the procedure for generating a heterogeneous cancer sample that consists of different subclones.** The eight subclones are related to each other according to a clonal ancestor tree. Each node in the tree represents a genetically different clone. The two lines represent the diploid genome with heterozygous and homozygous mutations. The germline mutations are assigned to the normal sample which builds the root of the tree. All nodes in the tree inherit the set of mutations from the parent and receive new cancerous mutations. The green bars illustrate that the clones exist at different frequencies in the tumor.

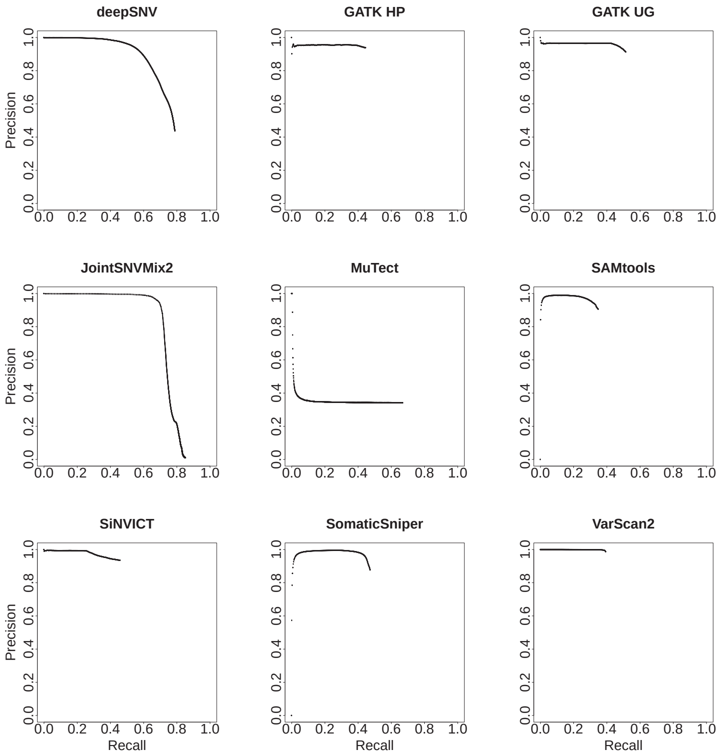

Additional file 1: Figure B: **Precision-recall curves for all tools.** This plot displays the precision and recall curve for each tool as described in Additional file 1: Section C. The whole set of predictions of the callers varies between 107, 952 for VarScan2, and 560, 428 for MuTect. JointSNVMix2 is the outlier, as it outputs more than 19 million variants. In this plot, we show every $500th$ point, and in the case of JointSNVMix2 every $2'000th$.

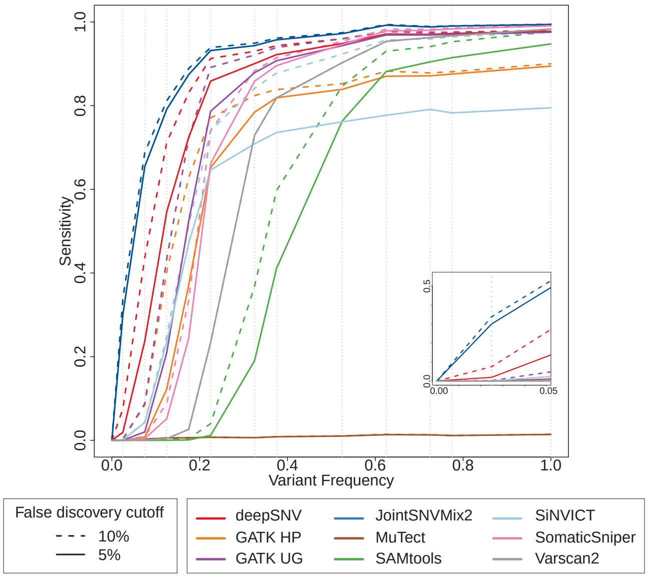

Additional file 1: Figure C: **Sensitivity of the variant callers when considering only regions with sufficient coverage**. Similar to Figure 2A, this figure shows the sensitivity of the tools as a function of the variant allele frequency, but here, the ground truth variant set was reduced to only those variants that are in regions of sufficient coverage. That is, the regions in which the tumor and the normal bam file both have a coverage of at least 25×. JointSNVMix2 reaches a sensitivity of $\geq$ 96% for variants with frequency $\geq$ 0.35, and detects 99% of all clonal variants.

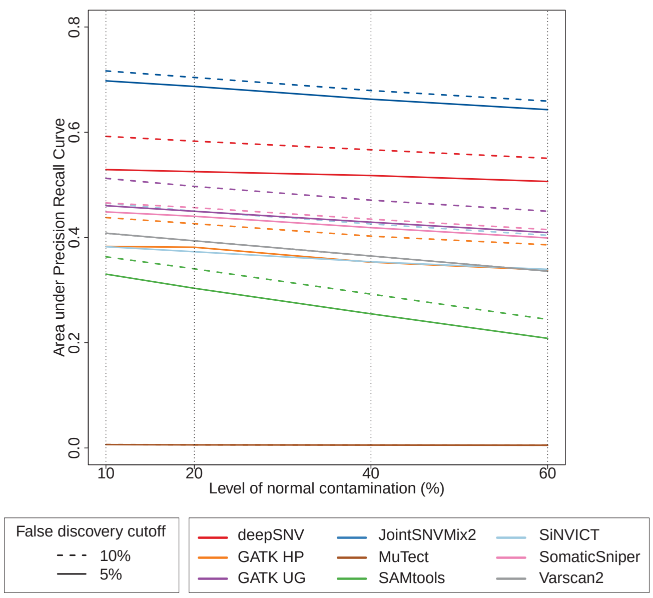

Additional file 1: Figure D: **Increasing fraction of DNA fragments from non-cancer cells.** We assessed the performance of the tools as a function of increasing levels of normal contamination. Similar as in Figure 2, the area under precision recall curve is shown with the two cutoffs for the false discovery rate $\alpha = 0.05$, and $\alpha = 0.1$ (see Additional file 1: Section C). The normal contamination corresponds to the four different levels (10%, 20%, 40%, and 60%) displayed in Figure 1 in step 5. More precisely, the specified percentage of normal reads was mixed into the tumor reads. As expected, the tools generally loose power in detecting variants with more normal contamination.

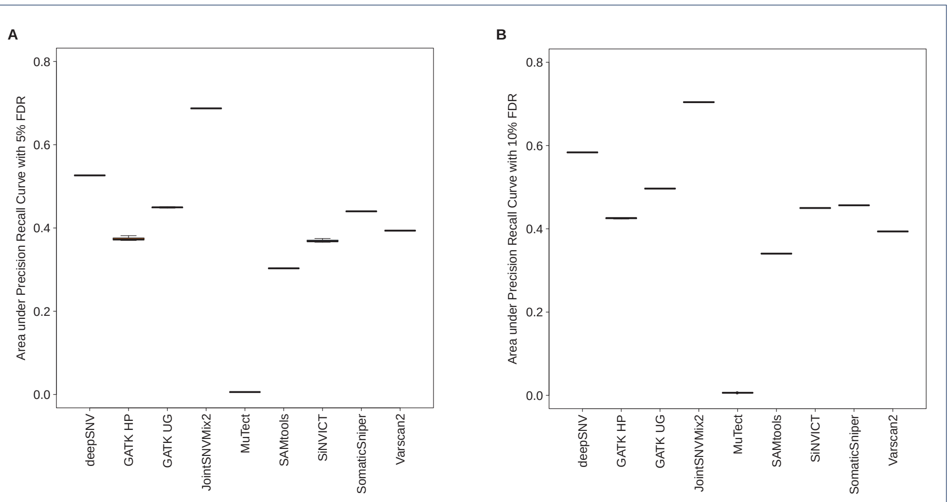

Additional file 1: Figure E: **The performances of the tools when repeating ten subsamplings of the entire alignment.** As illustrated in Figure 1, different levels of coverage were chosen (step 5). In order to assess the variance of the performance of the tools, the following procedure was repeated ten times: Subsampling 50% of the complete alignment file, running the tools and evaluating the area under precision-recall curve (auPRC). Depicted here are the boxplots of the auPRC when restricting the false discovery rate to 5% (panel **A**) and 10% (panel **B**). The performances clearly are very robust.

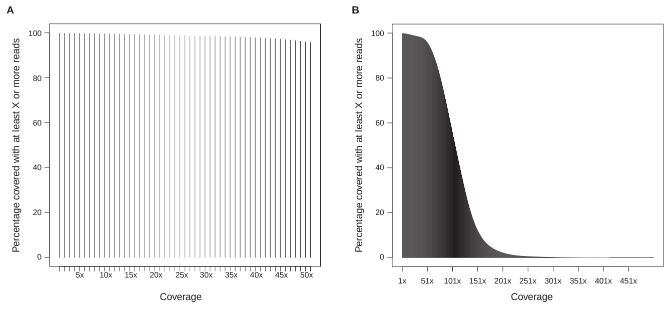

Additional file 1: Figure F: **Coverage profile.** The tools Qualimap [43], samtools [10], and BEDTools [44] were used to obtain coverage information such as mean and median coverage of the targeted regions. Also, the percentage of the targeted regions that is covered with at least x reads for $x \in \{1, 2, ..., 51\}$ (panel **A**), and $x \in \{1, 2, ..., 451\}$ (panel **B**) was computed. This is displayed here for the 50%-coverage bam file of the more sensitive alignment. Clearly, the coverage profile shows that the overall coverage is very good. The median coverage was 106×, and the mean coverage was 109×.

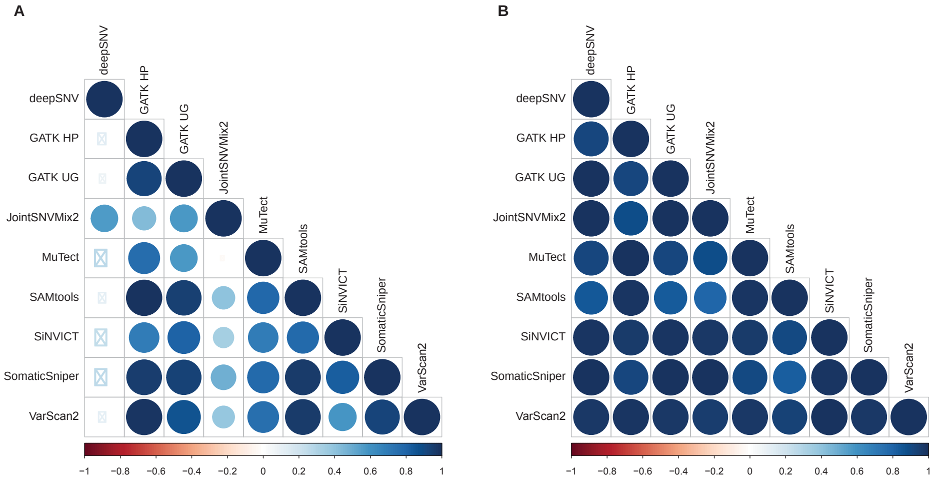

Additional file 1: Figure G: **Correlation of error profiles.** The error sources of false positive and false negative calls of the tools are shown in Figure 3. Here, the correlation of these error profiles between tools with the more sensitive alignment is depicted. **A** Correlations of the profiles for false positive calls. DeepSNV and JointSNVMix2 are generally less correlated with the other tools, since they clearly show a different false positive error profile. For instance, unlike for all other tools, the category *in normal* is not the largest. **B** Correlations of the profiles for false negative calls. The tools are all very correlated in their false negative error profile. The categories *low coverage* and *low coverage normal* are major error sources for all of them.

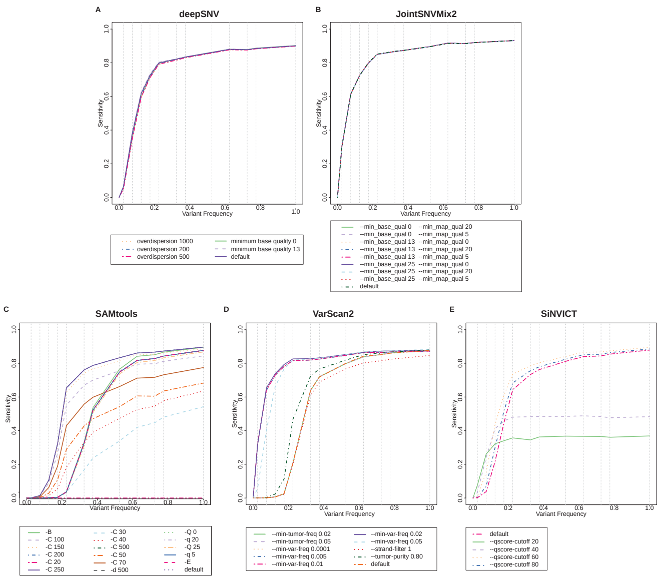

Additional file 1: Figure H: **Performance of deepSNV, JointSNVMix2, SAMtools, SiNVICT and VarScan2 when varying parameters of the tools**. As reported in Section "Parameter optimization of variant callers", different parameter settings where assessed for these tools. In Figure 4B, the performances of only a subset of these parameters are depicted. Here, for each tool, the performance of the tools with all different options that were tested are shown. The sensitivity is for the prediction set with at least 90% precision. **A** deepSNV. Varying the parameter for overdisperion or the threshold for the base quality does not affect the performance substantially. **B** JointSNVMix2. Different thresholds for the base and mapping quality do not influence the performance. This can be attributed to the statistical model of JointSNVMix2 as described in Section "Discussion". **C** SAMtools. The performance is very heterogeneous when using different parameters. Some of the curves are not visible, because they are identical to other curves. More precisely, the options `-C 200` and `-C 250` lead to basically identical performances. Furthermore, the performances with the options `-C 500`, `-E`, `-d 500`, `-q 20`, `-q 5`, and `-Q 0` are basically identical to the default run, which is why not all of these curves are visible here. **D**. VarScan2. The sensitivitiy of the default run can be improved substantially by reducing the threshold for the variant allele frequency. The options `--min-tumor-freq 0.02`, and `--min-tumor-freq 0.05` reach the same performance as the default. Also, the curves for `--min-var-freq 0.01`, `--min-var-freq 0.005`, and `--min-var-freq 0.0001` fall on the same line. **E** SiNVICT. Different thresholds for the q-score are evaluated with the parameter `--qscore-cutoff`. The best performance is reached with a threshold of 60.

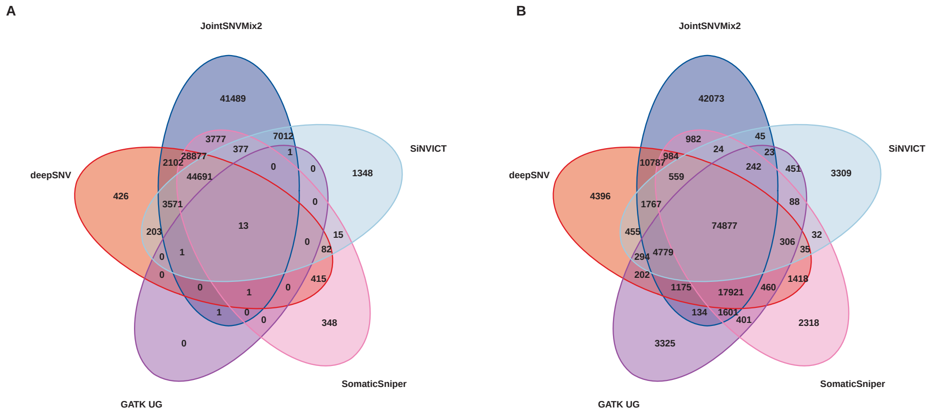

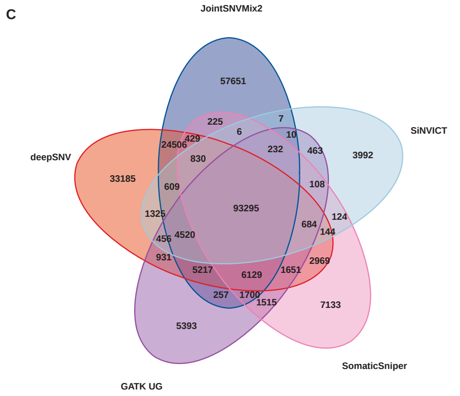

D

Evaluation of the overlap among the five tools			
FDR of individual mutation lists	Number of mutations	Precision	Recall
1%	13	100.0%	0.0%
5%	74,877	99.7%	34.4%
10%	93,295	99.6%	42.8%

Additional file 1: Figure I: **Venn diagrams displaying the number of shared variant calls between the top five callers from the default run in default mode**. The set of variants in the intersection was computed by comparing the positions of the variants, i.e. chromosome and nucleotide position. **A** The prediction set with at most 1% false discovery rate (FDR). The top predictions of the top five callers do not match. **B** The prediction set with at most 5% FDR. **C** The prediction set with at most 10% FDR. **D** The precision and recall of the sets of variants that are detected by all five tools with the three different FDR cutoffs. Clearly, many variants are missed when relying only on the set of variants detected by all five tools.

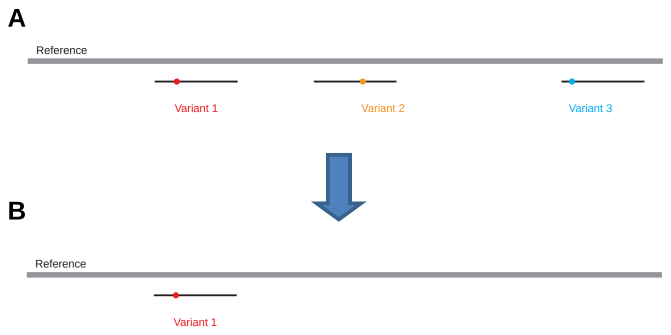

Additional file 1: Figure J: **Cartoon depicting the multi-mapper problem.** This cartoon assists in motivating our procedure for generating the ground truth variant set, which is described in Section "Simulation" and, in more detail, in Additional file 1: Section B. **A** A read that contains a variant may align equally well to three different locations in the reference genome. Each of the three alignments have, for instance, one mismatch compared to the reference, and hence obtain identical alignment scores. **B** One of the alignments is chosen to be the primary alignment, and the others, if they are reported in the bam file, are flagged as secondary alignment.

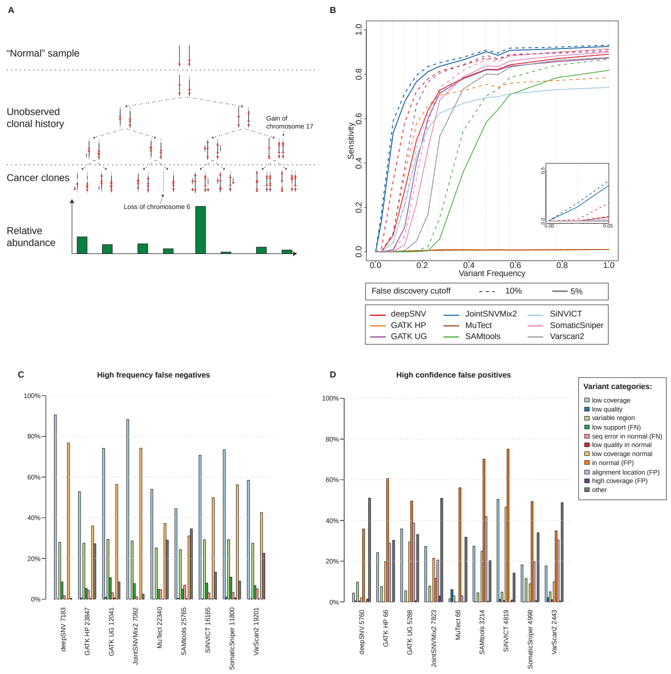

Additional file 1: Figure K: **Data simulation with CNVs and aneuploidy and subsequent evaluation of tools.** As specified in the original simulation which is depicted in Additional file 1: Figure A, the eight subclones are related through a clonal ancestor tree. Each node is a genetically different clone. The two lines represent the diploid genome. A copy number loss is indicated by a grey part of the line, instead of the black line, whereas a copy number gain is illustrated as an additional black line next to the two main alleles. The detailed description of this simulation can be found in Additional file 1: Section F. **B** Sensitivity of variant callers as a function of the variant allele frequency (VAF). Analogous to Figure 2A, the two false discovery cutoffs 10% and 5% are chosen. In Figure 2A, the sensitivities for the VAF intervals with effectively more than 3,000 ground truth variants are shown. Hence, the sensitivities here are also displayed for the intervals with at least 3,000 variants. **C** The categories of false negative variant calls, which have a ground truth VAF ≥ 0.25. The definitions of the categories remain the same as in Figure 3 and are explained in Section "Analysis of error sources". **D** The proportions of error sources for false positive calls, which are within the high confidence set of the variant calls. More precisely, the set of variants with at least 95% precision.

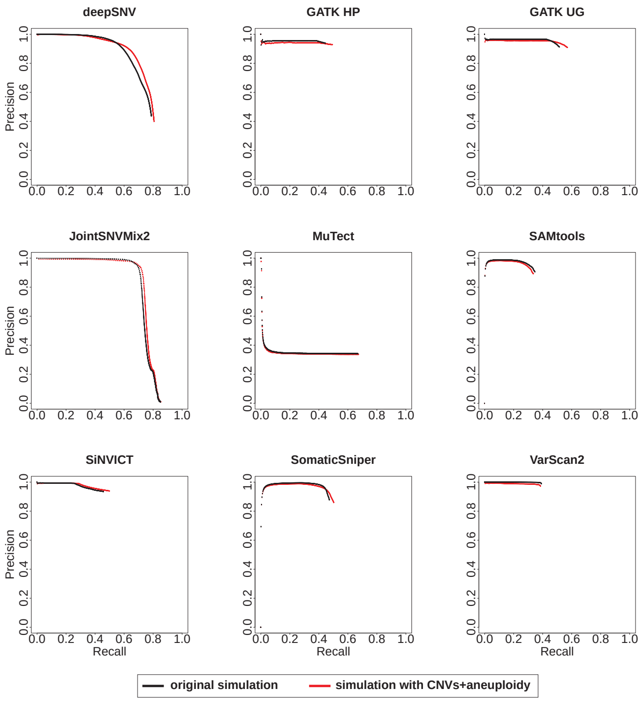

Additional file 1: Figure L: **Precision-recall curves for all tools from both simulations.** This plot displays the precision and recall curve for each tool. The black curve is the performance from the original simulation as also shown in Additional file 1: Figure B, and the red curve is the performance from the new simulation that includes CNVs and aneuploidy.

Tables

Additional file 1: Table S1: The performance of the ten best `rank-combinations` when comparing the area under precision recall curve (auPRC) with all predictions up to a false discovery rate of 5% and 10%, respectively. The `rank-combination` of tools that yields the best performance at the 10% level is in bold. As described in the main text, for the `rank-combinations`, the best tested versions of each tool were taken. More precisely, deepSNV and MuTect with the binomial filter, SAMtools with option `-C 200`, SiNVICT with the parameter `--qscore-cutoff 60`, VarScan2 with option `--min-var-freq 0.02`, and the default versions of JointSNVMix2, GATK HP, GATK UG, and somaticSniper.

`Rank-combination` **of which tools**	**auPRC 5% FDR**	**auPRC 10% FDR**
**deepSNV, JointSNVMix2, MuTect, SiNVICT, VarScan2**	0.7535	0.7759
deepSNV, JointSNVMix2, MuTect, SAMtools, VarScan2	0.7559	0.7746
deepSNV, JointSNVMix2, MuTect, VarScan2	0.7486	0.7731
deepSNV, JointSNVMix2, MuTect, somaticSniper, VarScan2	0.7494	0.7731
deepSNV, GATK UG, JointSNVMix2, MuTect, VarScan2	0.7536	0.7722
deepSNV, GATK HP, JointSNVMix2, MuTect, VarScan2	0.7555	0.7720
deepSNV, JointSNVMix2, MuTect, SAMtools, SiNVICT, VarScan2	0.7491	0.7713
deepSNV, JointSNVMix2, MuTect, somaticSniper, SiNVICT, VarScan2	0.7436	0.7709
deepSNV, GATK HP, JointSNVMix2, MuTect, SiNVICT, VarScan2	0.7502	0.7701
deepSNV, GATK HP, JointSNVMix2, MuTect, somaticSniper, VarScan2	0.7473	0.7696
deepSNV, GATK HP, JointSNVMix2, MuTect, SAMtools, VarScan2	0.7506	0.7692

Additional file 1: Table S2: The number of shared variant calls and the auPRC$_{90}$ as a measure of their performance of all subsets of size two of the nine variant callers. For the pairwise intersections, the best tested versions of each tool were taken. More precisely, deepSNV and MuTect with the binomial filter, SAMtools with option `-C 200`, SiNVICT with the parameter `--qscore-cutoff 60`, VarScan2 with option `--min-var-freq 0.02`, and the default versions of JointSNVMix2, GATK HP, GATK UG, and somaticSniper. The set of variants in the intersection was computed by comparing the positions of the variants, i.e. chromosome and nucleotide position. The variants which are shared between two callers can be ranked according to the confidence score of either tool. For this reason, we always indicate which tools' score was used for ranking in the third column.

Which tools	Number of shared variant calls	Which tools' score for ranking	auPRC 10% FDR
deepSNV, GATK HP	91169	deepSNV	0.4181
deepSNV, GATK UG	106177	deepSNV	0.4818
deepSNV, JointSNVMix2	214786	JointSNVMix2	0.6821
deepSNV, MuTect	138204	deepSNV	0.6133
deepSNV, SAMtools	93005	deepSNV	0.4268
deepSNV, SiNVICT	106235	deepSNV	0.4872
deepSNV, somaticSniper	100343	deepSNV	0.4415
deepSNV, VarScan2	137550	deepSNV	0.6321
GATK HP, GATK UG	122680	GATK HP	0.4360
GATK HP, JointSNVMix2	133048	JointSNVMix2	0.4486
GATK HP, MuTect	84418	MuTect	0.3847
GATK HP, SAMtools	113184	SAMtools	0.4026
GATK HP, SiNVICT	97866	SiNVICT	0.4336
GATK HP, somaticSniper	111905	somaticSniper	0.4040
GATK HP, VarScan2	118442	VarScan2	0.4428
GATK UG, JointSNVMix2	154235	JointSNVMix2	0.5136
GATK UG, MuTect	92529	MuTect	0.4192
GATK UG, SAMtools	127507	SAMtools	0.4489
GATK UG, SiNVICT	110993	SiNVICT	0.4844
GATK UG, somaticSniper	132758	somaticSniper	0.4648
GATK UG, VarScan2	132409	VarScan2	0.4950
JointSNVMix2, MuTect	141587	JointSNVMix2	0.6165
JointSNVMix2, SAMtools	137994	JointSNVMix2	0.4556
JointSNVMix2, SiNVICT	124385	JointSNVMix2	0.5168
JointSNVMix2, somaticSniper	145978	JointSNVMix2	0.4709
JointSNVMix2, VarScan2	204924	VarScan2	0.6926
MuTect, SAMtools	82839	SAMtools	0.3792
MuTect, SiNVICT	94017	SiNVICT	0.4287
MuTect, somaticSniper	84029	somaticSniper	0.3835
MuTect, VarScan2	124210	VarScan2	0.5686
SAMtools, SiNVICT	100112	SAMtools	0.4408
SAMtools, somaticSniper	124457	SAMtools	0.4468
SAMtools, VarScan2	120131	VarScan2	0.4467
SiNVICT, somaticSniper	102100	somaticSniper	0.4517
SiNVICT, VarScan2	111450	VarScan2	0.5025
somaticSniper, VarScan2	121563	SomaticSniper	0.4524

Additional file 1: Section B (Data simulation)

This section explains how the variant call format (VCF) file containing the ground truth germline and somatic mutations was generated, and how the somatic mutations were assigned to cancer subclones. In order to obtain a set of germline and cancer mutations as close to reality as possible, the tumor and matched normal data from a clear cell renal cell carcinoma (ccRCC) was used. One frozen primary clear cell renal cell carcinoma and its matched normal tissue from a 78-year-old ccRCC patient were obtained from the tissue biobank of the Institute of Surgical Pathology at the University Hospital Zurich. Hematoxylin and eosin stained tissue sections were reviewed by a pathologist specialized in uropathology. The tumor tissue contained 90% tumor cells, and was classified as tumor stage 1b (International Union against Cancer; 7th edition), and tumor differentiation grade 2 (2016 World Health Organisation/International Society for Uropathology) [45]. DNA was

extracted from frozen sections of the tumor and normal tissues using the Blood and Tissue Kit (Qiagen). The exome was sequenced using the Illumina HiSeq 2000 system. The paired 100-base reads from the tumor and matched normal sample were clipped and trimmed using Trimmomatic [46], and aligned with bowtie2 using the options `--very-sensitive` and `-k 20`, such that for each read, up to 20 valid alignments are reported. PCR duplicates were removed using Picard [47].

The motivation for the choice of the ground truth variant set is as follows. If a read maps to several positions equally well, the aligner decides randomly which one of the alignments is declared as the primary alignment. In order to deliver reproducible results, the aligner makes this decision in a deterministic way, such that, if one would align the same reads again, the same alignment would be destined as the primary alignment. Additional file 1: Figure J illustrates this procedure. In panel A, a read aligns equally well to three positions with one mismatch in each case. Panel B represents the decision of the aligner, to declare the first one as the primary alignment. The red mismatch would potentially be called as a variant, even though it could also be a false positive. Now, if one takes all the variants from primary alignments as ground truth and generates reads from this simulated sample with subsequent alignment, the aligner would select the same alignment as primary, i.e. the one with the red mutation in the cartoon. Since this was declared as ground truth before, it would be counted as a true positive. Therefore, the number of false positives due to multi-mappers would be underestimated. In order to avoid this, the following procedure was done to obtain the ground truth VCF.

From the bam files, two different versions were generated for tumor and normal sample: one with all reported alignments, i.e. up to 20 per read, which were all declared as primary alignment, and one without any ambiguous multi-mappers. More precisely, for the latter bam file, for each read that maps to several positions, only the most reliable mapping was kept. If a read had two or more equally good mappings it was discarded because one cannot be sure which alignment is the correct one. The variant caller freebayes [42] was run on these four bam files using the following parameters: `min_alternate_count=4`, `min_alternate_fraction=0.01`, `pooled_continuous=‘‘pooled_continuous’’`, `min_mapping_quality=0`. Freebayes is a tool that does not jointly analyze tumor and normal bam file, hence it was run on the four bam files separately.

The two lists of variants (VCFs) from the tumor and normal bam files with all multi-mappers contain many variants that are false positives due to misalignment. Conversely, the two VCFs from the tumor and normal bam files without any multi-mappers contain only variants that are either true positives or that arose from different error sources. By subtracting the latter set of variants from the former set of variants, one obtains lists of variants that are only due to uncertain multi-mappers. The goal was to mix these two types of variants in a way to most accurately portrait the real cancer sample.

We observed that the median number of alignments for a multi-mapper was 3. At most one of the three alignments can be the correct one, and only at most one alignment would remain, after a filter for mapping quality or only keeping the one which is flagged as primary alignment. Therefore, we chose to take 25% of the

mutations that are due to uncertain multi-mappers to mix into the variants that are either true positives or due to other sources of errors. The obtained set of variants from the normal sample was declared as set of ground truth germline mutations. The obtained set of mutations from the tumor sample was intersected with the normal mutations in order to filter out the germline mutations. The remaining tumor variants were declared as ground truth tumor varianst for the simulation.

Subsequently, the two generated germline and somatic ground truth VCF files are used to create reference genomes for the different subclones of the artificial cancer sample. The mutations in the two files are assigned to nodes in a balanced tree containing eight leaves as depicted in Additional file 1: Figure A. In order to do so, we traverse the two files simultaneously and select the mutations in sorted order according to their position in the genome. For each mutation we first assign it uniformly, at random, to one tree level and in a second step uniformly at random to a node of that level. In a third step, we decide if the variant is homozygous with a chance of 50% or heterozygous with a chance of 50% for each parent. If a variant contradicts an already inserted variant in any node of the sub-tree having the selected node as the root we discard it. Otherwise we place it in the sub-tree. A total of 217,507 somatic and 456,680 germline mutations found in the real RCC and matched normal sample were distributed on the phylogenetic tree. In the final step, the mutations are integrated into a reference genome. For each node two references are generated, one for each parent. With the help of Wessim [37], reads for each of the artificial references were created, where the number of created reads per file depends on the frequency of the sub-clone in the tumor tissue. More precisely, 50 million was taken as 100%, and then the following percentages of reads for the eight cancer clones were generated: 6.5%, 9.2%, 3.2%, 3.5%, 5.7%, 0.7%, 6.2%, 64.6%. This was determined by drawing a Dirichlet-distributed random vector. Finally, the reads were merged into one file. The subsamplings of the complete alignment file, which are indicated in Figure 1 step 5, were done using Picard [47].

Additional file 1: Section C (Evaluation)

Area under precision-recall curve In Additional file 1: Figure B the precision-recall curves are shown for all tools without any cut-off. All variants are considered in the ranking according to the score or p-value of the respective tool, which reflects the confidence of the caller that the variant call is correct. For MuTect, this means that we take first all variants with the label "PASS", and afterwards all the ones with "REJECT" in a random order. The area under the precision-recall curve (auPRC) is in general higher if the lists contain additional low-confidence predictions. This becomes evident when comparing the precision-recall curves of, for instance, JointSNVMix2 and VarScan2 in Additional file 1: Figure B. Since we do not want to penalize tools that report only high-confidence variants by default, we truncate all predictions to have a precision of at least 90% and 95%, respectively, before we compute the auPRC. The truncation however is not trivial because the precision is not a smooth function of the score. We obtain a numerically stable measurement by taking the median truncation cutoff from 50 bootstraps.

Additional file 1: Section D (Model description)

In this section, we describe the statistical models of the variant callers. To facilitate the comparison between the models, we introduce common nomenclature for variables and map the formulas from the original articles to this nomenclature as far as possible. The definition of variables is given in Additional file 1: Table S3.

Additional file 1: Table S3: **Variable definitions**

V	set of potential variants. We skip the index of the variant v whenever this is clear from the context to keep the notation uncluttered.
N	identifier for normal sample (the identifier is omitted if not necessary).
T	identifier for tumor sample (the identifier is omitted if not necessary).
D_i	read data for sample $i \in \{T, N\}$ for a given genomic location. We skip the index of the genomic location whenever this is clear from the context.
c_i	coverage depth for sample $i \in \{T, N\}$ at a given genomic location.
$c_{v,i}$	number of reads supporting allele $v \in V$ in sample i.
$f_{v,i}$	$\frac{c_{v,i}}{c_i}$ if $c_i > 0$, 0 otherwise; the variant allele frequency for $v \in V$.
h_i	genotype in sample i: 0=homozygous reference, 1=heterozygous variant, 2=homozygous for variant.
e_i	$e_i \in [0,1]^{c_i}$ are the probabilities of erroneous base calls for each read covering the current position.
R	$R \in \{A, C, G, T\}$ is the reference base at the current position.
B	$B \in \{A, C, G, T\} \setminus \{R\}$ is an alternate allele at the current position.

deepSNV The tool deepSNV [24] models each possible variant allele $v \in V$ at a given genomic position independently. In order to avoid strand bias, each strand is treated separately. It is tested, whether the frequency of v has changed between samples. Since the true variant frequency is unknown, deepSNV models the variant frequency in sample i with a beta distribution with mean $f_{v,i}$ to account for overdispersion. Taking the uncertainty about the true variant frequency into account, the likelihood of observing read data D_i is given by

$$P(D_i \mid \alpha) = g(c_{v,i}; c_i, f_{v,i}, \alpha),$$

where $g(\cdot; c, f, \alpha)$ is the probability density function of the beta-binomial distribution with c trials, mean f and variance $cf + c^2 \frac{f}{\alpha}$. The parameter α can be estimated from the data:

$$\hat{\alpha} = \operatorname*{argmax}_{\alpha \in \mathbb{R}_+} \prod_{v \in V} g(c_{v,N}; c_N, f_{v,N}, \alpha) \cdot g(c_{v,T}; c_T, f_{v,T}, \alpha).$$

A p-value is computed using the likelihood ratio test (χ^2-test with one degree of freedom) for the test statistic

$$S_v = -2 \ln \frac{\max_f \left[g(c_{v,N}; c_N, f, \hat{\alpha}) \cdot g(c_{v,T}; c_T, f, \hat{\alpha}) \right]}{\max_{f_N} g(c_{v,N}; c_N, f_N, \hat{\alpha}) \cdot \max_{f_T} g(c_{v,T}; c_T, f_T, \hat{\alpha})}.$$

By default, deepSNV only takes into account bases with base quality 25 or higher.

GATK UnifiedGenotyper (HP), GATK HaplotypeCaller (UG) The GATK provides two different variant callers, the HaplotypeCaller (GATK HP), and the UnifiedGenotyper (GATK UG). The HaplotypeCaller employs local de novo assembly of haplotypes to detect SNVs and indels at the same time [27]. This feature is expected to make it more accurate when calling variants in regions with increased variability. Both tools use a Bayesian approach [25, 26], which computes the posterior probability for all ten possible diploid genotypes (AA, AC, AG, AT, CC, CT, CG, TT, TG, GG) via the Bayes rule

$$P(h \mid D) = \frac{P(D \mid h)P(h)}{P(D)},$$

where the likelihood of the data factorizes into

$$P(D \mid h) = \prod_{j=1}^{c_T} P(b_j \mid h),$$

and b_j is the jth base at the current position. The probability of a base b given an allele B can be written as

$$P(b \mid B) = \begin{cases} \frac{e}{3} & \text{if } b \neq B \\ 1 - e & \text{if } b = B. \end{cases}$$

The genotype with the greatest posterior probability is then chosen.

JointSNVMix2 The model of JointSNVMix2 [28] is similar to the deepSNV model. Instead of testing whether the data in the tumor and normal sample can be explained by the same allele frequency, JointSNVMix2 computes the posterior probability of the joint-genotype, which is modeled as a multinomial variable. That is, it does not assume that there is just one true genotype. In a diploid genome, the set of possible genotypes is $h \in \{0, 1, 2\} =: G$, and therefore, the set of possible joint-genotypes consists of $G \otimes G = \{(h_N, h_T) : h_T, h_N \in G\}$. In contrast to deepSNV, the statistical model of JointSNVMix2 incorporates base and mapping quality. Hence the uncertainty of base calls and alignment is included. In a preceding training step, JointSNVMix2 calculates the MAP estimates of the parameters of the beta-binomial model via the EM algorithm. In the default prior distribution, it is assumed that most of the probability mass is located at the homozygous reference case. We used the parameter `post_process` [48], which computes the post-processed probability that the variant is somatic based on a classification method. This parameter is recommended because it was shown to be more accurate than the standard probabilities [48].

MuTect Like deepSNV, MuTect [29] detects variant alleles independently for a given site. A Bayesian classifier is employed to identify variants in the tumor sample. More precisely, MuTect compares the variant model to the null model, which explains all non-reference bases by sequencing errors or contamination from the normal tissue and other individuals. An alternative allele $v \in V$ becomes a candidate

variant if the log-likelihood ratio between null model and variant model exceeds the predefined threshold θ_T, which is based on the expected mutation frequency:

$$LOD_T(v) = \log_{10}\left(\frac{P(D_T \mid e, v, R, f_v)}{P_{f_{cont}}(D_T \mid e, v, R)}\right) \geq \theta_T.$$

Here, f_{cont} is the expected fraction of wrong bases due to contamination, which is estimated by the tool ContEst [49]. The likelihood of the data factorizes into the probabilities for each observed base b at the current position, which is defined as

$$P(b \mid e, v, R, f_v) = \begin{cases} f_v \frac{e}{3} + (1 - f_v)(1 - e) & \text{, if } b = R \\ f_v(1 - e) + (1 - f_v)\frac{e}{3} & \text{, if } b = v \\ \frac{e}{3} & \text{, otherwise.} \end{cases}$$

After computing the list of candidate variants, several filters are applied to remove likely false positives. These filters consider, for example, the mapping quality, the strandedness or the proximity to indels.
Finally, the remaining variants are classified as somatic or germline mutations using a second Bayesian classifier. This time, two different models are weighted against each other: the null model, which assumes the candidate variant is a heterozygous germline mutation, and the variant model, which predicates that the candidate variant is not present in the normal sample. The candidate variant is classified as somatic, if this ratio exceeds a threshold θ_N, which is based on the expected mutation frequency and the expected frequency of a germline mutation:

$$LOD_N(v) = \log_{10}\left(\frac{P_{f_v=0}(D_N \mid e, v, R)}{P_{f_v=0.5}(D_N \mid e, v, R)}\right) \geq \theta_N.$$

Variants are reported without confidence information or a score.

SAMtools At a given position, the SAMtools [10] model takes into account three possible genotypes: homozygous for the reference, heterozygous variant, or homozygous variant. Based on the observed reads and their associated error probabilities, it computes the posterior probability of the homozygous reference case. To this end, the genotype likelihood is computed as

$$P(D \mid h) = \frac{1}{2^c}\prod_{j=1}^{l}((2-h)(1-e_j) + he_j) \cdot \prod_{j=l+1}^{c}((2-h)e_j + h(1-e_j)),$$

where it is assumed that the first l ($l \leq c$) bases match the reference allele, and the rest of the bases match the alternate allele. The variant quality, which is used to determine whether a site is a variant or not, is defined as

$$Q_{var} = -10\log_{10} P(h = 0 \mid D, \Phi).$$

Φ consists of the prior probabilities for the genotypes. It is assumed that most sites are homozygous for the reference allele.

SiNVICT The method of SiNVICT [30] was designed to be able to handle data sets with ultra-deep coverage and low tumor purity, as is the case especially when deep-sequencing the circulating tumor DNA from a blood sample of a cancer patient. In order to call mutations, SiNVICT uses a Poisson model, in which the p-value to distinguish bona fide mutations from errors is computed as

$$p = 1 - e^{-\lambda} \sum_{i=0}^{c_{v,T}-1} \frac{\lambda^i}{i!},$$

where $\lambda = c_T \cdot r$, and r is the error rate of the sequencing platform. The confidence score, or q-score, is then defined as $Q = -10 \log_{10} p$. In a second Poisson model, SiNVICT distinguishes mutations as germline or somatic, by assessing whether the observed number of reads that support the mutation at a location can be explained by a heterozygous germline mutation. Finally, SiNVICT outputs all variants which pass the threshold for the q-score, and separates them into six lists, which correspond to filters at various stringency levels. These levels filter according to minimum read depth, degree of strand-bias, average position of the mutation on the reads, signal-to-noise ratio, and proximity to homopolymer regions.

SomaticSniper Similar to the approach from the GATK tools, the statistical model of SomaticSniper [31] takes into account all possible ten diploid genotypes (AA,AC,AG,AT,CC,CT,CG,TT,TG,GG) at a given position. In contrast to the GATK tools, somaticSniper jointly analyzes the data from the tumor and normal sample. The somatic score, which reflects the confidence of the variant call, is calculated as

$$S = -10 \log_{10} \frac{\sum_{h_T = h_N} [\mathbb{P}(D_N \mid h_N) \cdot \mathbb{P}(D_T \mid h_T) \cdot \mathbb{P}(h_T \mid h_N) \cdot \mathbb{P}(h_N)]}{\sum_{h_1} \sum_{h_2} \mathbb{P}(D_N \mid h_1) \cdot \mathbb{P}(D_T \mid h_2) \cdot \mathbb{P}(h_2 \mid h_1) \cdot \mathbb{P}(h_1)}.$$

The probabilities for the genotype in the tumor given a genotype in the normal are fixed *a priori*. Afterwards, the variants are filtered according to predefined thresholds for quality, coverage, etc.

VarScan2 At each position that meets quality standards, VarScan2 [32] decides on the genotype h_T and h_N in tumor and normal sample independently based on the read counts.

If $h_T \neq h_N$, a Fisher's exact test is performed to evaluate the read counts in both samples and to classify the candidate variant. More precisely, if the p-value is not significant, the candidate variant is categorized as germline. If the p-value is significant, the decision for the category of the variant is summarized in Additional file 1: Table S4.

If $h_T = h_N$, the candidate variant is categorized as germline. For the germline variants, a Fisher's exact test is performed which assesses the possibility that the candidate variant can be attributed to a sequencing error. Only the significant variants are kept. Finally, a set of filters is applied which remove potential false positives due to artifacts generated during the sequencing and alignment process.

Additional file 1: Table S4: **Classification of variants in the case of a significant p-value. (LOH indicates loss of heterozygosity)**

h_T	h_N	category
0	0	germline
	1	LOH
	2	unknown
1	0	somatic
	1	germline
	2	discarded
2	0	somatic
	1	LOH
	2	germline

Additional file 1: Section E (Commands of the tools)

In this section we provide code snippets to show how the tools where ran in default mode. The entire code that was used to run the simulation, the tools, and the evaluation can be found here [33].

deepSNV The deepSNV method is implemented in an R-package. The R version `3.3.0` and the deepSNV version `1.18.1` was used. For each chromosome $i \in \{1, 2, \ldots, 22, X, Y\}$, deepSNV was run separately as `deepSNV_TU_NO[[i]] = deepSNV(test=tumor.bam, control=normal.bam, alternative = ‘‘two.sided’’, model=‘‘betabin’’, regions=all_exons_list[[i]])`, where `all_exons_list[[i]]` is a GRanges object that contains all targeted regions of the respective chromosome. Afterwards, the deepSNV object is converted into a table that lists all detected SNVs with `summary_deepSNV_TU_NO_all[[i]] = summary(deepSNV_TU_NO[[i]], sig.level=`$1 - 10^{-7}$`, adjust.method=NULL)`. Finally, the summary tables of all chromosomes are concatenated and the p-values are corrected for multiple testing using the following command: `p.adjust(summary_all$p.val, method="BH", n)`, where `n` is the number of tests performed. In our analysis, no p-value cutoff is employed, but the p-values are simply used to obtain a ranking of the variants. To make the output of deepSNV comparable to the other SNV callers, all single-nucleotide indels are removed from the predictions, since the other callers do not report them. Also, deepSNV detects variants, where the variant allele frequency in the normal sample is higher than in the tumor sample. These are loss of heterozygosity (LOH) events and are filtered out as well, because we focus on somatic SNVs in this study.

GATK HaplotypeCaller The GATK HaplotypeCaller is a tool that also outputs germline mutations. Therefore, it was run on the tumor and normal bam files separately, and then the variants which where only found in the tumor file where considered somatic variants. Bowtie2 assigns a mapping quality of 255 for reads that align uniquely. However, GATK filters out reads with mapping quality of 255 by default. Therefore, we add an optional argument that tells GATK to reassign the mapping quality of 255 to 254: `/pathTo/jre1.7.0_40/bin/java -jar /pathTo/GenomeAnalysisTK-3.3-0/GenomeAnalysisTK.jar -R reference_genome_hg19 -T HaplotypeCaller -I tumor.bam -o outputFile.vcf -rf ReassignOneMappingQuality -RMQF 255 -RMQT 254`. The same command was used for the normal bam file with `-I normal.bam`.

Afterwards, all variants from the tumor vcf file that where also in the normal vcf file were filtered out as germline mutations.

GATK UnifiedGenotyper The GATK UnifiedGenotyper commands were identical to the GATK HaplotypeCaller commands, except that `-T UnifiedGenotyper` was used. The germline variants were also filtered out, again by running the same procedure for the normal bam file, and then keeping the tumor only variants.

JointSNVMix2 In a first step, JointSNVMix2 learns the parameters for the statistical model via the EM-algorithm with the following command: `/pathTo/bin/jsm.py train --skip_size 150 --model beta_binomial --priors_file /pathTo/JointSNVMix-0.8-b2/config/beta_binomial.priors.cfg --initial_parameters_file /pathTo/JointSNVMix-0.8-b2/config/beta_binomial.params.cfg reference_genome_hg19 normal.bam tumor.bam outputFile_Paratemers.txt`. The option skip-size indicates that every 150th position is used for estimation. This is necessary to limit the amount of memory that is used. In the second step of JointSNVMix2, the variants are called with `/pathTo/bin/jsm.py classify --model beta_binomial outFile_SNVs.txt --parameters_file outputFile_Paratemers.txt --post_process reference_genome_hg19 normal.bam tumor.bam`. The `--post_process` parameter is recommended and computes the post-processed posterior probability of a somatic mutation by using feature-based classification [48].

MuTect The following command was used to run MuTect: `java -Xmx8g -jar /pathTo/MuTect-1.1.4/muTect-1.1.4.jar -T MuTect --input_file:normal normal.bam --input_file:tumor tumor.bam -R reference_genome_hg19 --out outputFile.txt -vcf outputFile.vcf --coverage_file outputFile_coverage.txt`. The file outputFile.vcf then contains all variants.

SAMtools With "SAMtools", we refer to the `samtools/bcftools` suite. Similarly to the GATK HaplotypeCaller and UnifiedGenotyper, SAMtools calls both, germline and somatic variants. Therefore, as for the two GATK tools, SAMtools was run on tumor and normal bam files separately, and then those variants were considered as somatic that were only detected in the tumor file. The command for the tumor file was `/pathTo/samtools-1.2/samtools mpileup --output-tags DP4,DP -uf reference_genome_hg19 tumor.bam | /pathTo/samtools-1.2/bcftools/bcftools-1.2/bcftools call -mv -Oz > outputFile.vcf.gz`, and the command for the normal bam file was analogous.

SiNVICT First, the tumor and normal bam files were processed with the tool `bam-readcount` [50] to obtain statistics for each location about the alleles and their qualities: `/pathTo/bin/bam-readcount -w 1 -f reference_genome_hg19 bam_file.bam -l bedfile.bed > readcount.out`. Next, SiNVICT was run with `/pathTo/sinvict/sfu-compbio-sinvict-1f69cc7/sinvict -t`

/pathTo/readcount/ -o /pathTo/sinvict_out/. These commands were run on the tumor and normal bam files separately. Finally, each tumor file output from SiNVICT was filtered, such that all mutations which are also in the normal file are removed.

SomaticSniper The command for SomaticSniper was /pathTo/somatic-sniper/build/bin/bam-somaticsniper -F vcf -f reference_genome_hg19 tumor.bam normal.bam outputFile.vcf with SomaticSniper version 1.0.2.3-4-gcfecd4b. The somatic score provided by somaticSniper was used to rank the SNVs.

VarScan2 The input for VarScan2 are the pileup files from the tumor and normal bam files. These are generated with samtools and the reference genome as samtools mpileup -f reference_genome_hg19 tumor.bam > tumor.pileup, and analogously for the normal pileup file. Variants are called with java -Xmx4g -jar /pathTo/varscan2/VarScan.v2.3.7.jar somatic normal.pileup tumor.pileup outputFile.txt. Finally, VarScan2 provides the processSomatic command, which divides the output into germline, somatic and LOH events with java -Xmx4g -jar /pathTo/varscan2/VarScan.v2.3.7.jar processSomatic outputFile.txt.snp. In this study, we focus on the somatic SNVs and do not take the germline or LOH variants into account.

Additional file 1: Section F (Data simulation with CNVs and aneuploidy)

The set of genetic variations in a real tumor sample also includes CNVs as well as aneuploidy. In the main study, these genomic changes were excluded, since the focus was the analysis of the performance of SNV callers. While CNVs and aneuploidy also play an important role in tumor evolution, the only effect they would have on SNVs is to influence the variant allele frequency. For instance, if a gene that harbors a mutation undergoes a copy number gain, the relative allele frequency of the variant would increase, and vice versa for a copy number loss. However, in order to demonstrate that CNVs and aneuploidy do not change the conclusions made here, we assessed it by creating another simulated data set, in which CNVs and aneuploidy were introduced in addition to the SNVs and indels. The procedure of the simulation and evaluation is described in the following part of this section.

Similar to the SNVs and indels, a tool for detecting CNVs, namely Control-FREEC [51], was used to detect the CNVs in the real ccRCC sample. Control-FREEC identified 12 CNVs, including nine copy number losses and three copy number gains. Similar to the SNVs and indels, the CNVs were distributed randomly on the phylogenetic tree. All nodes in the tree inherit the CNVs from the parent and possibly receive new CNVs. Additionally, two aneuploidies were introduced at random. More precisely, one of the subclones was selected to have lost chromosome six, and another subclone was selected to have gained chromosome 17, which is inherited by its children in the tree. An illustration of the hereby obtained phylogenetic tree can be found in Additional file 1: Figure K panel A. As in the original analysis, all nodes in the tree inherit the set of mutations from the parent.

The SNVs and indels remain the same as in the original simulation, but now, mutations which a node receives as new cancerous mutations, may also fall into a copy number gain region, and mutations, which are in a copy number loss region are removed. If a clone undergoes a copy number gain, the mutations in this region are in the gained part as well. Similar to the original analysis, the variants were placed into the genomes using the software library SeqAn [36], and reads were generated using Wessim [37]. The proportions of the eight cancer clones were determined to be 11.7%, 45.7%, 19.7%, 1.3%, 12.9%, 2.3%, 0.8%, 5.2% by once drawing a Dirichlet-distributed random vector. The green bars in Additional file 1: Figure K panel A illustrate that the clones exist at different frequencies in the tumor. The procedure of aligning, and subsequent processing of the bam files, as well as variant calling was as in the original analysis. This time, only one alignment, coverage level, and contamination level was chosen. More precisely, the more sensitive alignment was done, as described in Section "Variant calling pipeline", the coverage level was set to 50%, the contamination level to 20%, and all variant callers were run in default mode as described in Additional file 1: Section E. That way, the results depicted in Additional file 1: Figure K panels B, C, and D, are comparable to Figures 2A, 3B, and 3D. Additional file 1: Figure K panel B shows the sensitivity as a function of the variant allele frequencies (VAF). The VAF intervals at which the sensitivity is diplayed have on average $17,329$ variants. The one with ground truth VAFs in the interval $[0.45, 0.5)$ has with $5,091$ the least number of ground truth variants. This might explain the slightly nosier performance in this range, where some variant callers have a decrease of $0\% - 2\%$ in their sensitivity when compared to the next interval with VAFs in $[0.5, 0.55)$. Overall, the performance is very similar to the one from the original analysis. The proportions of the various categories of false negative and false positive calls are depicted in Additional file 1: Figure K panels C and D. As in the original analysis, the major sources of false negative calls are *low coverage*, *low coverage in normal*, and *variable region*. Concerning the false positive calls, we note that *in normal*, *low coverage*, *low coverage in normal*, as well as *alignment location* are still the most prominent sources of errors. In addition, the new precision-recall curves were examined in comparison to the original precision-recall curves. Both curves are displayed in Additional file 1: Figure L. The curves from both simulations are very similar. Minor variations in performance are expected, since the new simulation has some effects on the number of variants and their frequencies. The total number of variants is now $212,249$ instead of formerly $217,507$, due to copy number losses. Moreover, the number of variants with certain VAFs has changed. For instance, we now have $25,122$ less variants with a VAF below 0.1. And in total, we have now 514 different ground truth VAFs, as compared to 268 in the original simulation. It is noticeable that all tools but GATK HP, SAMtools, and VarScan2 perform even slightly better in the simulation that includes CNVs and aneuploidy. The reduced number of low frequency variants might explain the mild increase in performance of variant callers. In general, the conclusions from the original analysis remain unchanged in light of the new simulation with CNVs and aneuploidy.

Additional file 1: Section G (Displaying sensitivity as a function of frequencies)

The ground truth list contains variants with 268 different variant allele frequencies (VAFs). In order to obtain a smooth sensitivity profile for Figures 2A, 4A, B, C, as well as Additional file 1: Figure C, and Additional file 1: Figure H, we merge the variants into VAF bins of size 0.05. That is, we compute the sensitivity for all variants with ground truth VAF in the following intervals $(0, 0.05), [0.05, 0.1), \ldots, [0.95, 1.0), [1.0, 1.05)$, respectively. The last interval only contains clonal variants, which have a VAF of 1.0. Finally, in order to avoid displaying noise, only the sensitivity of the bins which have at least 200 variants are plotted. These were bins with $3,404 - 47,305$ variants. Since each bin represents an interval, the sensitivity that belongs to this bin is plotted over the middle of the interval. For instance, the sensitivity for the all variants with VAF in $(0, 0.05)$ is plotted at 0.025. The grey dotted vertical lines in these figures indicate these positions. Figures in this manuscript were generated using the R language of statistical computing [52] including the R packages RColorBrewer, gplots, corrplot and VennDiagram.

Additional file 1: Section H (Integration of variant callers: `rank-combination`)

To combine the scores of the several different variant callers into a single combined score, we treat the problem analogously to combining the p-value from several independent experiments. That combination may be performed by simply taking the product (Fisher's method) or in a variety of other ways compared for example in [53]. The main difficulty is that the scores of the variant callers are neither equally accurate p-values nor, being derived from a common set of alignments, independent. To remove the problem of directly comparing the score of different callers, we simply use the rank with 1 being the top score and so on downwards. Taking N to be the size of the union of the variants discovered by the different callers, we map the rank of the variants to the interval $[0, 1]$ with the transformation

$$p_i = \frac{r_i}{2(N+1)}$$

where r_i is the rank of a variant from caller i. We omit a subscript labelling the variant. The ranked variants are therefore lined up starting near 0 for the top variant, while unranked variants in the union are placed at $\frac{1}{2}$.

To treat the problem of dependence between the different callers, we standardize the correlations between them. The rationale behind this is clear from the extreme case of placing two copies of the same caller into the scores to be combined. A straightforward product weights the duplicated caller much more heavily, even though the duplication adds no new information. Similarly, similar callers provide less information than more disparate ones, and standardizing the correlations makes the combination more equal.

To perform the standardization we first transform from the hypercube to the entire space using the cdf Φ of a normal distribution

$$x_i = \Phi^{-1}(p_i)$$

so that unranked variants are mapped to the origin. Then we find the matrix of second moments

$$\mathrm{E}\left[\boldsymbol{x}\boldsymbol{x}^{\mathrm{T}}\right] = C$$

averaged over the N variants. Taking D to be a diagonal matrix whose elements are the diagonal of C, the corresponding correlation form is then

$$\tilde{C} = D^{-\frac{1}{2}} C D^{-\frac{1}{2}}$$

For the example using deepSNV, JointSNVMix2, MuTect, SiNVICT, and VarScan2, we have the matrices

$$1000C = \begin{pmatrix} 61 & 42 & 58 & 54 & 60 \\ 42 & 58 & 53 & 52 & 49 \\ 58 & 53 & 863 & 82 & 74 \\ 54 & 52 & 82 & 87 & 62 \\ 60 & 49 & 74 & 62 & 102 \end{pmatrix}, \text{and}$$

$$1000\tilde{C} = \begin{pmatrix} 1000 & 701 & 251 & 736 & 761 \\ 701 & 1000 & 239 & 731 & 646 \\ 251 & 239 & 1000 & 299 & 250 \\ 736 & 731 & 299 & 1000 & 654 \\ 761 & 646 & 250 & 654 & 1000 \end{pmatrix}.$$

From the correlation matrix $\tilde{C}$ we create a standardized version $\tilde{S}$ by replacing all of its off-diagonal elements by their average. We also map back to the second moment space $S = D^{\frac{1}{2}} \tilde{S} D^{\frac{1}{2}}$, which for our example gives

$$1000\tilde{S} = \begin{pmatrix} 1000 & 527 & 527 & 527 & 527 \\ 527 & 1000 & 527 & 527 & 527 \\ 527 & 527 & 1000 & 527 & 527 \\ 527 & 527 & 527 & 1000 & 527 \\ 527 & 527 & 527 & 527 & 1000 \end{pmatrix}, \text{and}$$

$$1000S = \begin{pmatrix} 61 & 31 & 121 & 39 & 42 \\ 31 & 58 & 117 & 37 & 40 \\ 121 & 117 & 863 & 145 & 156 \\ 39 & 37 & 145 & 87 & 50 \\ 42 & 40 & 156 & 50 & 102 \end{pmatrix}.$$

The reason we retain the diagonal elements of C in S is that their scales represent how far from the origin the original p_i were and how many variants were unranked. This is information we wish to retain and not standardize.

Next we transform to an equally correlated space

$$\chi_i = S^{\frac{1}{2}} C^{-\frac{1}{2}} x_i$$

where we perform the matrix powers using an eigenvalue decomposition. The matrix of second moments is now S with equal correlations

$$\mathrm{E}\left[\boldsymbol{\chi}\boldsymbol{\chi}^{\mathrm{T}}\right] = S^{\frac{1}{2}} C^{-\frac{1}{2}} \mathrm{E}\left[\boldsymbol{x}\boldsymbol{x}^{\mathrm{T}}\right] \left(C^{-\frac{1}{2}}\right)^{\mathrm{T}} \left(S^{\frac{1}{2}}\right)^{\mathrm{T}} = S^{\frac{1}{2}} C^{-\frac{1}{2}} C C^{-\frac{1}{2}} S^{\frac{1}{2}} = S$$

because the matrices are symmetric. Finally, we map back to the hypercube

$$\rho_i = \Phi(\chi_i)$$

to obtain the desired p-value like coordinates with equalised correlations. A simple product of the ρ_i for each variant provides a combined score from all the different callers which is finally ranked.

Of course, this procedure combines the output from several different variant callers as evenly as possible. As shown in Section "Performance with default parameters", the performance of different callers varies widely and if one knew the relative performance on real data one would, of course, wish to weight the better callers more heavily.
